# Single-cell and bulk transcriptomic datasets enable the development of prognostic models based on dynamic changes in the tumor immune microenvironment in patients with hepatocellular carcinoma and portal vein tumor thrombus

**DOI:** 10.3389/fimmu.2024.1414121

**Published:** 2024-10-28

**Authors:** Wangxia Tong, Jieyue Zhong, Qiuyan Yang, Han Lin, Bolun Chen, Tao Lu, Jibing Chen, Ning Luo

**Affiliations:** ^1^ Department of Hepatology, Ruikang Hospital Affiliated to Guangxi University of Chinese Medicine, Nanning, Guangxi, China; ^2^ Department of Hepatobiliary Surgery, Ruikang Hospital Affiliated to Guangxi University of Chinese Medicine, Nanning, Guangxi, China; ^3^ Center for Translational Medicine of Integrated Traditional Chinese and Western Medicine, Ruikang Hospital Affiliated to Guangxi University of Chinese Medicine, Nanning, Guangxi, China; ^4^ Department of Neurology, RuiKang Hospital Affiliated to Guangxi University of Chinese Medicine, Nanning, Guangxi, China

**Keywords:** single-cell RNA-seq, hepatocellular carcinoma, portal vein tumor thrombosis, immune characteristic, tumor microenvironment

## Abstract

**Background:**

Hepatocellular carcinoma (HCC) patients exhibiting portal vein tumor thrombosis (PVTT) face a high risk of rapid malignant progression and poor outcomes, with this issue being compounded by a lack of effective treatment options. The integration of bulk RNA-sequencing (RNA-seq) and single-cell RNA-seq (scRNA-seq) datasets focused on samples from HCC patients with PVTT has the potential to yield unprecedented insight into the dynamic changes in the tumor microenvironment (TME) and associated immunological characteristics in these patients, providing an invaluable tool for the reliable prediction of disease progression and treatment responses.

**Methods:**

scRNA-seq data from both primary tumor (PT) and PVTT cells were downloaded from the Gene Expression Omnibus (GEO) database, while the International Cancer Genome Consortium (ICGC) and Cancer Genome Atlas (TCGA) databases were used to access bulk RNA-seq datasets. scRNA-seq, clustering, GSVA enrichment, mutational profiling, and predictive immunotherapeutic treatment analyses were conducted using these data with the goal of systematically assessing the heterogeneity of PT and PVTT cells and establishing a model capable of predicting immunotherapeutic and prognostic outcomes in patients with HCC.

**Results:**

These analyses revealed that PVTT cells exhibited patterns of tumor proliferation, stromal activation, and low levels of immune cell infiltration, presenting with immune desert and immune rejection-like phenotypes. PT cells, in contrast, were found to exhibit a pattern of immunoinflammatory activity. Core PVTT-associated genes were clustered into three patterns consistent with the tumor immune rejection and immune desert phenotypes. An established clustering model was capable of predicting tumor inflammatory stage, subtype, TME stromal activity, and patient outcomes. PVTT signature genes were further used to establish a risk model, with the risk scores derived from this model providing a tool to evaluate patient clinicopathological features including clinical stage, tumor differentiation, histological subtype, microsatellite instability status, and tumor mutational burden. These risk scores were also able to serve as an independent predictor of patient survival outcomes, responses to adjuvant chemotherapy, and responses to immunotherapy. *In vitro* experiments were used to partially validate the biological prediction results.

**Conclusion:**

These results offer new insight into the biological and immunological landscape of PVTT in HCC patients, By utilizing individual patient risk scores, providing an opportunity to guide more effective immunotherapeutic interventional efforts.

## Introduction

Primary liver cancers are frequently diagnosed throughout the globe, with hepatocellular carcinoma (HCC) being the most common pathological subtype of thereof, accounting for 75-85% of all cases (1, 2). In the early stages of tumorigenesis, HCC generally does not present with any distinct clinical symptoms such that 80% of HCC patients are only diagnosed when the tumor is already relatively advanced. As there are no reliable treatments available for advanced liver cancer, affected patients generally survive for 10-24 months after diagnosis (3, 4). The liver exhibits unique anatomical and biological characteristics that can facilitate the frequent invasion of the intrahepatic portal vein system by HCC tumors, leading to portal vein tumor thrombosis (PVTT) (5, 6). The incidence of HCC combined with PVTT has been reported to be up to 10-40% on initial diagnosis (7) and 44.0-62.2% on autopsy in Japan (8), and these patients face worse prognostic outcomes as compared to HCC patients unaffected by PVTT (9). These adverse outcomes include high rates of tumor invasion, portal hypertension resulting from PVTT, and insufficient hepatic reserve functionality (10). In patients with untreated PVTT, the median survival duration is just 4.0 months (6).

At present, the American and European guidelines for HCC patients exhibiting PVTT only recommend the use of targeted drug treatment (11, 12), whereas supplementary local therapy is recommended under Asian HCC patient guidelines. Recommended supplementary treatment options for these PVTT patients include hepatectomy, local radiotherapy, TACE, or hepatic arterial infusion chemotherapy (HAIC) (13–15). Despite the growing array of treatment options available to these HCC patients with PVTT, clinical data suggest that they continue to experience very poor survival outcomes (16–18). A high degree of heterogeneity is observed with respect to HCC patient responses to treatment owing to factors including the complexity of the tumor microenvironment, epigenetic differences, and the heterogeneous nature of the tumors themselves (17, 19, 20). This patient-specific heterogeneity needs to be taken into account to select the optimal treatment approach for each patient. Molecular biomarkers based on the genomic characteristics of a given tumor can offer insight into the dynamic changes in immune responses and tumor microenvironment composition within cancer patients, providing an invaluable resource for the production of disease progression and treatment responses (21, 22).

Advances in cancer genomics have led to the generation of extensive bulk transcriptomic sequencing (bulk RNA-seq) datasets that can provide insight into average levels of gene expression within a given sample. In contrast, single-cell RNA-seq (scRNA-seq) datasets can yield nuanced information regarding transcriptomic heterogeneity at the cellular level, allowing researchers to better understand gene expression distributions (23, 24). These unique advantages have prompted many researchers to explore HCC-associated biomarkers through the integration of bulk RNA-seq and scRNA-seq analyses, allowing for precise patient selection and stratification. Here, scRNA-seq and bulk RNA-seq data were leveraged to conduct systematic analyses exploring the immunological landscape of PVTT with the goal of clarifying the differences between primary tumor (PT) and PVTT tissue samples. Characteristic PVTT-associated genes were identified through a screening strategy and used to construct a model capable of predicting HCC patient prognosis, with two external validation cohorts then being used to successfully confirm the ability of this model to stratify patients based on risk. The association between this risk model and immune cell infiltration was also assessed, while the ability of risk score values to predict the progression of disease or HCC patient responses to treatment was also assessed. The corresponding molecular biology experiments were used to evaluate the bioinformatics findings. *In vitro* assays have proved that the prediction results of bioinformatics analysis are reliable. Together, the results of these analyses provide new information that may benefit future efforts to manage HCC patients in clinical settings.

## Materials and methods

### Data selection and preparation

The LIHC GSE149614 scRNA-seq dataset was obtained from the GEO (https://www.ncbi.nlm.nih.gov/) database. This dataset was comprised of PT tissue samples from 10 patients with LIHC, and PVTT tissue samples from 2 patients with LIHC. The scRNA-seq analyses were conducted with the R Seurat package (v 4.2.0) (25), transforming raw data into Seurat objects. The R Harmony package was employed to correct for batch effects. Any cells exhibiting the expression of more than 8,000 genes or less than 500 genes were excluded, as were cells in which more than 20% of the UMIs were derived from the mitochondrial genome. A linear regression model was used for the normalization of core cell gene expression, while ANOVAs were used to screen the top 2000 genes exhibiting a high degree of variability. Single-cell samples were subjected to principal component analysis (PCA), selecting the top 20 principal components (PCs) for further analyses. Overall dimensionality reduction analyses for the top 20 PC pairs were achieved with the “tSNE” and “UMAP” algorithms. The R singleR (26) package was used for the preliminary identification of cell types, with further cell type annotation being achieved with the CellMarker database (http://xteam.xbio.top) (27) and cell surface marker-related data. Cellular characteristics and core gene expression profiles in PVTT samples were not corrected for the effect of the cell cycle.

The TCGA database (https://portal.gdc.cancer.gov/) was accessed to obtain bulk RNA-seq data, clinical data, and SNP site data from the TCGA-LIHC cohort, which consisted of 374 LIHC samples together with 50 samples of normal tissue. Samples were excluded if they did not have complete survival information such that 370 LIHC samples were retained for the final analysis. Bulk RNA-seq data, clinical information, and SNP mutation site data of TCGA-LIHC were downloaded from the TCGA database. In addition, the ICGC database (https://dcc.icgc.org/) was accessed to obtain bulk RNA-seq data and corresponding clinical data for the ICGC-(LIRI-JP) cohort consisting of 243 LIHC tissue samples and 202 samples of normal tissue. ICGC data were utilized as an external dataset for model validation.

### Identification of PVTT-associated characteristic genes, pseudotime trajectory analyses, and correlation analyses

Characteristic genes in PVTT cell clusters and other clusters were detected with the Seurat package “FindAllMarkers” function through Wilcoxon rank sum tests, selecting marker genes for each cluster based on the following parameters: logFC=0.25, P<0.05. The R Monocle2 package (v2.8.0) was employed for pseudotime analyses of HCC cells, with the original processed count matrix data from Seurat serving as the input for this analysis. An expression family object was created with the Cell Data Set function, setting the lower limit of detection to 0.5. Cell developmental trajectories were explored through an unsupervised approach based on highly variable genes that were selected by Monocle. All parameters were set to the default values with the exception of the dispersion empirical parameter, which was set at 1. An unsupervised nonparametric gene set variation analysis (GSVA) (28) algorithm was conducted to assess pathway activity within PVTT cell clusters in comparison to other cell clusters. The Hallmark gene set was used for GSVA enrichment analyses, with the gene set generated by Luo et al (29). being used to analyze differences in biological processes when comparing the PVTT cell clusters to other clusters. This gene set included AT2, angiogenesis, epithelial-mesenchymal transition (EMT) markers, inflammation score, anti-inflammatory score, and antigen presentation. In addition, a gene set established by Chen et al. (30) associated with forms of cell death including necroptosis, ferroptosis, and autophagy was utilized. Genes associated with biological processes were stored, and the links between PVTT features and biological pathways of interest were probed through correlation analyses.

### Candidate gene screening and core gene selection

The R limma package was used to screen for differentially expressed genes (DEGs) in the TCGA and ICGC cohorts (p < 0.05 and |Log2FC|> 1), with candidate genes from these datasets being used to screen for characteristic prognosis-related genes through univariate Cox proportional risk regression analyses. The overlap between the characteristic PVTT-related genes from scRNA-seq analyses and the prognostic genes identified in the TCGA and ICGC datasets was established as the candidate gene set. ROC curves for these candidate genes in the TCGA and ICGC datasets were plotted with the R survROC (31) package to assess the ability of these candidate genes to predict the overall survival (OS) of patients with HCC. All genes with ROC values > 0.7 in the TCGA and ICGC datasets were selected, with the resultant gene list serving as the core gene list for predictive model development.

### Unsupervised core gene clustering and TME cell infiltration analyses

Patients were classified according to patterns of core gene expression based on an unsupervised clustering approach, with a consensus clustering algorithm being used to determine cluster numbers and stability with the ConsensuClusterPlus package and 1,000 repetitions to ensure classification stability (32). The R GSVA package was used to conduct GSVA enrichment analyses aimed at observing differences in biological processes when comparing established clustering models (28). GSVA analyses were performed with the Hallmark gene set, using an FDR < 0.05 as the cut-off.

The CIBERSORT package was used to assess the tumor immune microenvironment associated with different clusters, yielding insight into the infiltration status for 22 different immune cell types for each TCGA-LIHC patient sample. Correlations between immune cell infiltration and the core genes of interest were assessed based on Pearson correlation coefficients. Differences in immune cell infiltration, immune circulation scores, and tumor stroma scores were also assessed across different clusters with the IOBR package (33).

### Prognostic model development and validation

Regression coefficients (β) and expression levels for core genes were used to establish a prognostic risk scoring model for HCC patients with PVTT through a multivariate Cox regression approach as follows: HCC with PVTT. Risk score = gene exp1×β1+ gene exp2×β2+ gene exp3×β3. The optimal cut-off value was used to classify patients as low-risk or high-risk, and the survminer package was then used to compare the survival curves for patients in these two risk groups. ROC curves and area under the curve (AUC) values were used to assess model specificity and sensitivity based on the prediction of HCC patient OS at 1, 2, and 3 years. Wilcoxon or Kruskal-Wallist tests were used to assess the relationship between clinicopathological features and survival, with the TCGA and ICGC datasets respectively being used for training and validation. Samples in different subgroups were analyzed based on the following classifications: age (≤ 60 or > 60 years), gender (male, female), grade (grade 1-2, grade 3-4), stage (stage 1-2, stage 3-4), T stage (T1-2, T3-4). To gain greater insight into the relationship between clinicopathological features and the survival of patients, stratified survival analyses of the low- and high-risk patients were conducted for these different clinicopathological subgroups. Multiple patient clinical features and risk scores were included in this effort, and the R cph function was used to generate a nomogram capable of predicting patient 1-, 2-, and 3-year OS. Calibration curves were employed for model validation.

### Predictive analyses of immunotherapy and chemotherapy sensitivity

The GEO database was accessed to download four immunotherapy-related datasets containing transcriptomic data and corresponding information regarding patient immunotherapy responses. The analyzed therapies in these datasets included the treatment of metastatic melanoma with pembrolizumab (GSE78220) (34), the treatment of advanced urothelial carcinoma with atezolizumab urothelial carcinoma (IMvigor210 cohort) (35), the treatment of renal cell carcinoma with nivolumab (GSE67501) (36), and pan-cancer tumor responses to the combination of PD-1 and CTLA-4-targeting therapies derived from the TCIA database (https://tcia.at/) (37). The oncoPredict package was also used to examine the potential relevance of risk scores to chemotherapy responses, yielding IC50 values for drugs included in the Genomics of Drug Sensitivity in Cancer (GDSC) (https://www.cancerrxgene.org/) (38) and the Cancer Treatment Response Portal (CTRP). Correlations between risk scores and the IC50 values for different drugs were employed for drug screening, after which differences in IC50 values for these drugs were compared between the low- and high-risk patient groups. The top 10 drugs exhibiting the highest sensitivity in the high-risk group were then extracted, and the results were presented with the R ggplot2 package.

### Cell lines and cell culture

Human hepatocellular carcinoma cell lines HepG2, Huh7 and Human Hepatic Stellate Cell line LX-2 were obtained from Pricella Life Science and Technology Co., Ltd. (#CL-0103, #CL-0120, #CL-0560, Wuhan, China). HCC-LM3 cell line was obtained from Haixing Biosciences Co., Ltd. (#TCH-C456, Suzhou, China). PLC/PRF/5 and MHCC-97-H cell lines were obtained from Ubigene Biosciences Co., Ltd. (#YC-C125 and #YC-A028, Guangzhou, China). All cell lines were cultured in DMEM medium (#PM150210) supplemented with 10% fetal bovine serum (#PM150210), and penicillin-streptomycin mix(#P1400) (all from Pricella Life Science and Technology Co., Ltd, Wuhan, China) at 37°C in a humidified incubator with 5% CO2.

### HCC patient samples and immunohistochemistry

The HCC tissues and para-tumor tissues (adjacent normal tissues; >2 cm from the tumors’ edges) were procured from the different three individuals undergoing liver cancer resection at the Department of Hepatobiliary Surgery, Ruikang Hospital Affiliated to Guangxi University of Traditional Chinese Medicine between December 2022 and October 2023.The studies were approved by the Ethics Committees of Ruikang Hospital Affiliated to Guangxi University of Traditional Chinese Medicine. The assigned ethical review approval number: KY2022-045 (**Supplementary Figure 4**). Immunohistochemistry (IHC) was performed according to standard protocols using PRR11, KIF11, RACGAP1, YY1, CREB1and SUZ12 antibodies. The information of antibodies are shown in **Supplementary Table 1**. Finally, the prepared liver tissue sections were photographed under a microscope (#BX53, Olympus, Shanghai, China) to evaluate the target staining and overall tissue morphology.

### Migration and Matrigel invasion assays

Matrigel matrix glue (#40183ES08, Yeasen Biotechnology, Shanghai, China) was diluted in a serum-free medium at 1:6, and 50ul was evenly spread into the upper chamber of Transwell chamber (#353097, BD Medical, Shanghai, China). The chamber was placed in a 24-well plate and incubated at 37°C for 4h to allow it to gelatinize. The cell density was adjusted to 2×10^5^ cells/mL, and 100μL per well was inoculated into the upper chamber of transwell chamber. The cells in the upper chamber were removed after 24h, fixed with methanol and 0.1% crystal violet (#G1063, Solarbio, Beijing, China) for 20min, stained for 10min, and washed twice with PBS. The cells were then counted under an inverted optical microscope (#BX53, Olympus, Shanghai, China).

### Cell viability assays

MTT Cell Proliferation and Cytotoxicity Assay Kit (S4025, Warbio, Nanjing, China) was used to measure the viability of transfected LM3 and HepG2 cells. Then cells were plated into 96-well plates(#1014000-T4, SAINING, Suzhou, China) at a density of 1×10^4^ cells per well and thereafter incubated for 24h, 48h and 72h, respectively. MTT solution (10 µL) was added to each well at the designated time intervals and incubated at 37°C for 2h. The absorbance at 570 nm was measured using a microplate reader (Infinite 200 PRO, Shanghai, China).

### Colony formation assay

HepG2 and LM3 cell suspension was prepared once the cell growth was in the logarithmic phase. The cells were then seeded into the six-well plates (#1010000-T4, SAINING, Suzhou, China) and incubated at 37°C, 5% CO2 and saturated humidity for two weeks. The culture was terminated if the clones visible to the naked eyes appeared in the dish. Afterward, the cells were rinsed with PBS twice and fixed with 5 mL of pure methanol (#BL539A, Ranjeck, Beijing, China) for 15 min. The methanol was then removed, and crystal violet ammonium oxalate solution (#G1063, Solarbio, Beijing, China) was added and incubated for 30 min. The staining solution was removed, and the sample was dried in air. Clones with more than 10 cells were counted using a light microscope (#BX53, Olympus, Shanghai, China). The colony formation rate was calculated by using the formula (number of clones)/(number of inoculated cells) × 100%.

### Western blotting analysis

HCC cells and tissues were lysed in radioimmunoprecipitation assay (RIPA) buffer (#R0020, Solarbio, Beijing, China) containing protease suppressors for 30 min. The protein concentration was quantified using a bicinchoninic acid (BCA) kit (#P0010S, Beyotime, Shanghai, China) in accordance with the manufacturer’s protocol. The total protein was subjected to heating at 95°C for 5 min. Thereafter, identical quantities of protein were separated by sodium dodecyl sulfate polyacrylamide gel electrophoresis (SDS-PAGE) (#P001A, Beyotime, Shanghai, China). The separated proteins were transferred to polyvinylidene fluoride (PVDF) (#FFP28, Beyotime, Shanghai, China) membranes after which electrometastasis was applied and then blocked with 5% skimmed milk at the room temperature for 60 min. The membranes were then stored overnight at 4°C with an appropriate primary antibody. The panel of antibodies used was shown in **Supplementary Table 1**. After washing 3 times in TBST buffer, the membranes were incubated at the room temperature for 1h with rabbit horseradish peroxidase (HRP)-conjugated secondary antibody (Goat, 1:10000, #BA12163708, Bioss, Beijing, China), then washed with the blocking solution and visualized by enhanced chemiluminescence (#6100, Clinx Science, Shanghai, China). Finally, quantity One gel analysis software was used to detect the signal intensity of each membrane. The intensity was measured relative to that of GAPDH.

### qRT-PCR

Total RNA was extracted from CRC cell lines using Monzol™ Reagent kit (#MI20201S, Mona, Suzhou, China) following the manufacturer’s instructions. cDNA was synthesized using a reverse transcription kit (#EG15133S, BestEnzymes, Lianyungang, China). The samples Glyceraldehyde-3-phosphate dehydrogenase (GAPDH) was used as an endogenous control. Each sample was prepared in triplicate. mRNA expression levels were calculated by comparing Ct method. qRT-PCR primers sequences were listed in **Supplementary Table 2**.

### Co-immunoprecipitation (Co-IP) assays

HepG2 and LM3 cells were first lysed according to the lysis method of adherent cells. After removal of non-specific binding, the samples were centrifuged at 2500rpm for 5 min, and the supernatant was used for subsequent immunoprecipitation. 2ug primary antibody used for immunoprecipitation was added and slowly shaken overnight at 4°C 20ul Protein A+G Agarose was added and slowly arose at 4°C for 1 h. The cells were centrifuged at 2500rpm for 5 min. Carefully remove the supernatant. Wash and precipitate with PBS for 5 times. 40ul of 1x SDS-PAGE electrophoresis buffer Vortex was added and the mixtures were obtained by high-speed centrifugation. Co-IP assays were performed according to Immunoprecipitation Kit with Protein A+G Agarose Gel (#P2197M, Beyotime, Shanghai, China) standard protocols.

### Establishment of transient transfected cell lines

The cells were transiently transfected with siRNAs, plasmids using Lipofectamine™ 3000 Transfection Reagent (#2773051, Thermo Fisher Scientific, Shanghai, China) according to the manufacturer’s instructions. PRR11, KIF11, RACGAP1, YY1, CREB1, SUZ12 cDNA were synthesized and cloned them into the pcDNA3.1(+) empty vectors by the GenePharma Technology Corp., The forward primer and reverse primer of KIF11, RACGAP1, PRR11, CREB1, YY1 and SUZ12 are shown in **Supplementary Table 4**. and sequencing was performed to verify the DNA sequence. The siRNA sequence of PRR11, KIF11, RACGAP1, YY1, CREB1, SUZ12 are shown in **Supplementary Table 3**.

### Statistical analysis

All experiments were performed in triplicates. Data are presented as the mean ± standard deviation. Statistical significance was determined using t-test for comparisons between two groups. R v 4.2.1 was used to conduct all statistical analyses, with *P* < 0.05 and FDR *q*-values < 0.05 being regarded as significant.

## Results

### scRNA-seq analyses of PVTT samples

**Figure 1:**
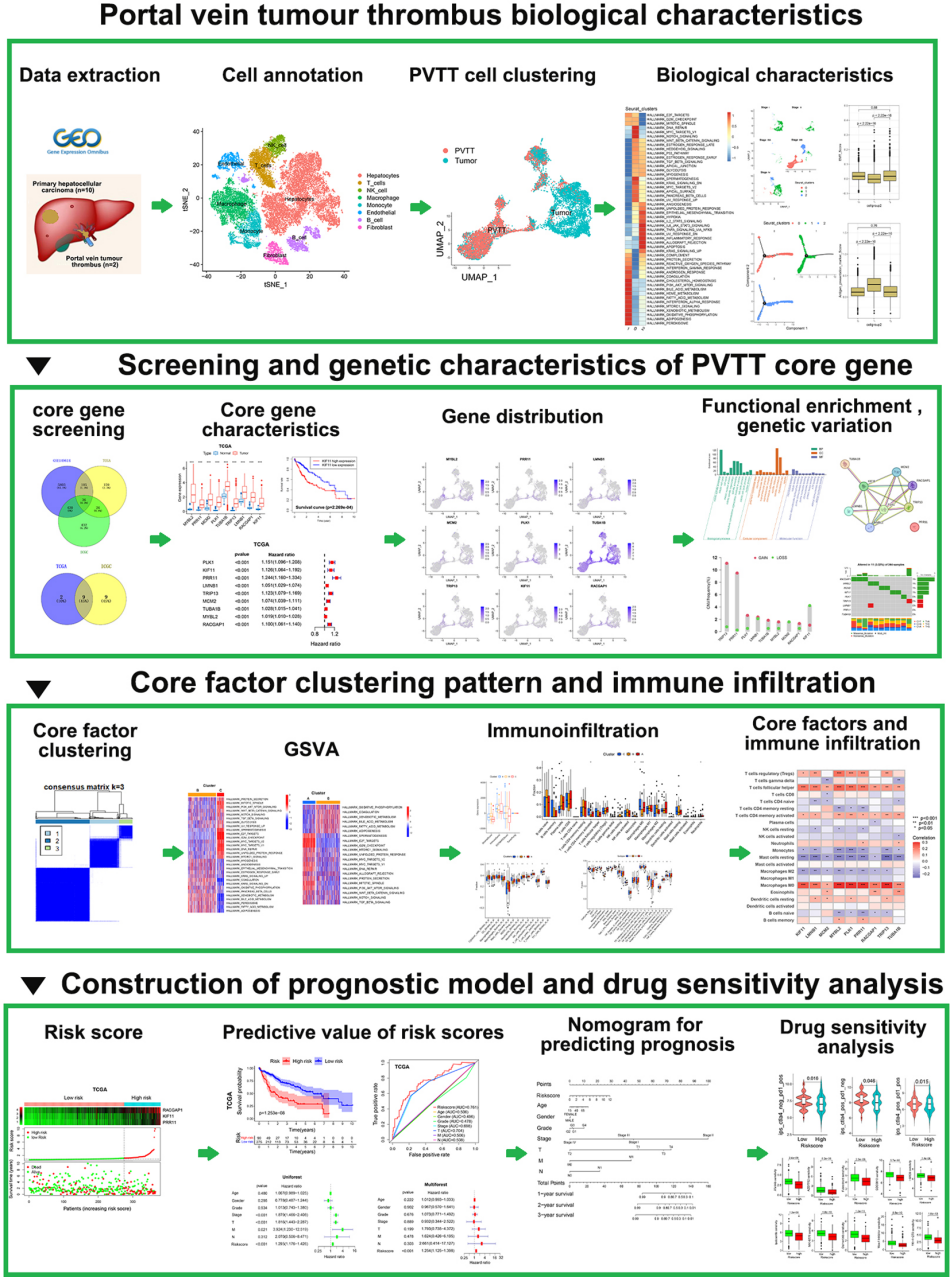
Study flowchart.

**Figure 2:**
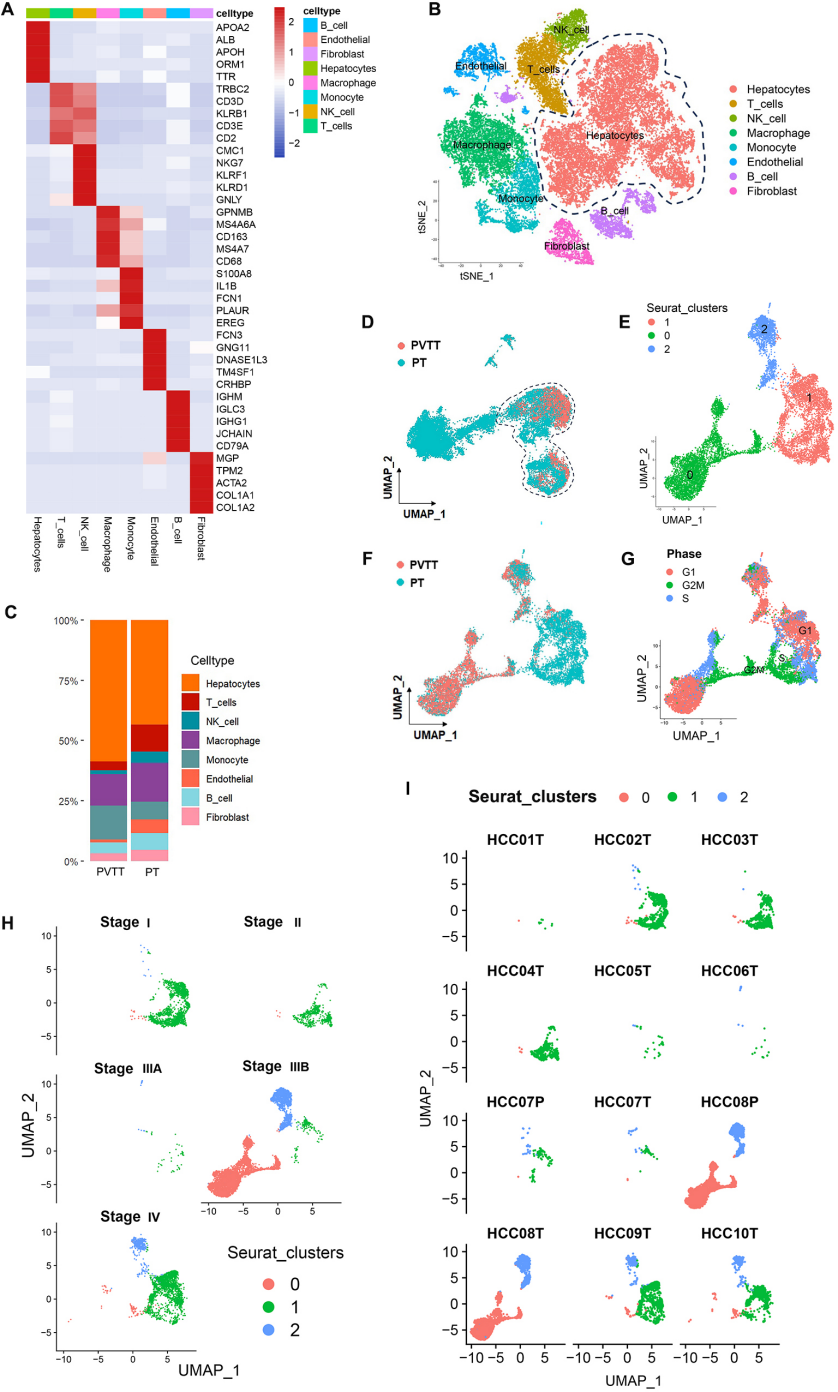
PVTT single-cell sources and distributions. **(A)** Marker genes were used to annotate cell types. **(B)** Eight cell types were identified in this dataset. **(C)** Proportion composition of cell types of in the PT and PVTT. **(D)**The clustering and grouping results for HCC cells. **(E)** The clustering and grouping results for the combination of PT and PVTT cells. **(F)** Clustering and grouping of the mixed PT and PVTT cells were performed to identify their sources and distributions. **(G)** The cell cycle phase for the mixed cells cluster from PT and PVTT samples; **(H)** The pathological stage for the mixed cells cluster from PT and PVTT samples; **(I)** The sample source stratification for the mixed cells cluster from PT and PVTT samples.

For an overview of the analytical approach for this study, see **Figure 1**. After acquiring data from the study performed by Lu et al (39). from the GEO database, scRNA-seq data for the included 10 PT and 2 PVTT tissue samples from the included HCC patients were analyzed. Following quality control screening, 40,384 cells were retained for analysis. Marker genes from the CellMarker database and source study were used to annotate cell types (**Figure 2A**), ultimately leading to the annotation of eight types of cells including hepatocytes, B cells, T cells, NK cells, endothelial cells, fibroblasts, monocytes, and macrophages (**Figure 2B**).

Further analyses of the hepatocyte cell subset were next conducted, with Copykat being used to conduct cellular CNV analyses of these hepatocytes, revealing that all hepatocytes in this CNV analysis were malignant tumor cells. Further analysis of the proportion of cell types indicated that PVTT contained a higher proportion of malignant hepatocytes. Compared with PT, the proportion of T cells, NK cells, endothelial cells, B cells and fibroblasts was decreased in PVTT. This indicated that the proliferation of malignant hepatocytes in PVTT is obvious, but it is also accompanied by the inhibition of tumor immunity and matrix activation (**Figure 2C**).

As cells of other subtypes beyond those found in PT samples may be present in PVTT samples, the cells in these PVTT samples were screened through two clustering and grouping strategies. Initially, all HCC cells were clustered and grouped, followed by the preliminary screening of the cell clusters from PVTT samples (**Figure 2D**). These screened clusters were then subjected to another round of clustering and grouping to identify those cell clusters consistent with PVTT-associated biological characteristics. This second round of clustering revealed that these cells were further stratified into three sub-clusters numbered 0, 1, and 2 (**Figure 2E**). Those cells in cluster 0 were derived from PVTT samples, whereas those from cluster 1 were derived from PT samples, and those from cluster 2 were from mixed PVTT and PT samples (**Figure 2F**). More specifically, cells in cluster 0 were primarily derived from samples T8, T9, and T10, together with low levels of input from samples T1-4 and T7. In contrast, cells in cluster 1 were primarily from samples T1-7 and T9-10. Cells in cluster 2 were primarily derived from samples T2-3 and T6-10 (**Figure 2I**). Cell cycle distribution analyses indicated that all three clusters contained cells in the S, G1, and G2M phases, with HCC cells in the G2M stage clustering at the junction of cells in clusters 0 and 1 (**Figure 2G**). Based on pathological staging data, patients in clusters 0 and 2 exhibited stage IIIB and IV disease, whereas cells in cluster 1 were associated with patients with disease stages spanning from I-IV (**Figure 2H**). These data suggest that while PVTT primarily developed in patients with advanced (stage IIIB-IV) HCC, tumor cells with PVTT-like characteristics are evident within PT tissue samples even when collected from patients with early-stage primary HCC.

**Figure 3:**
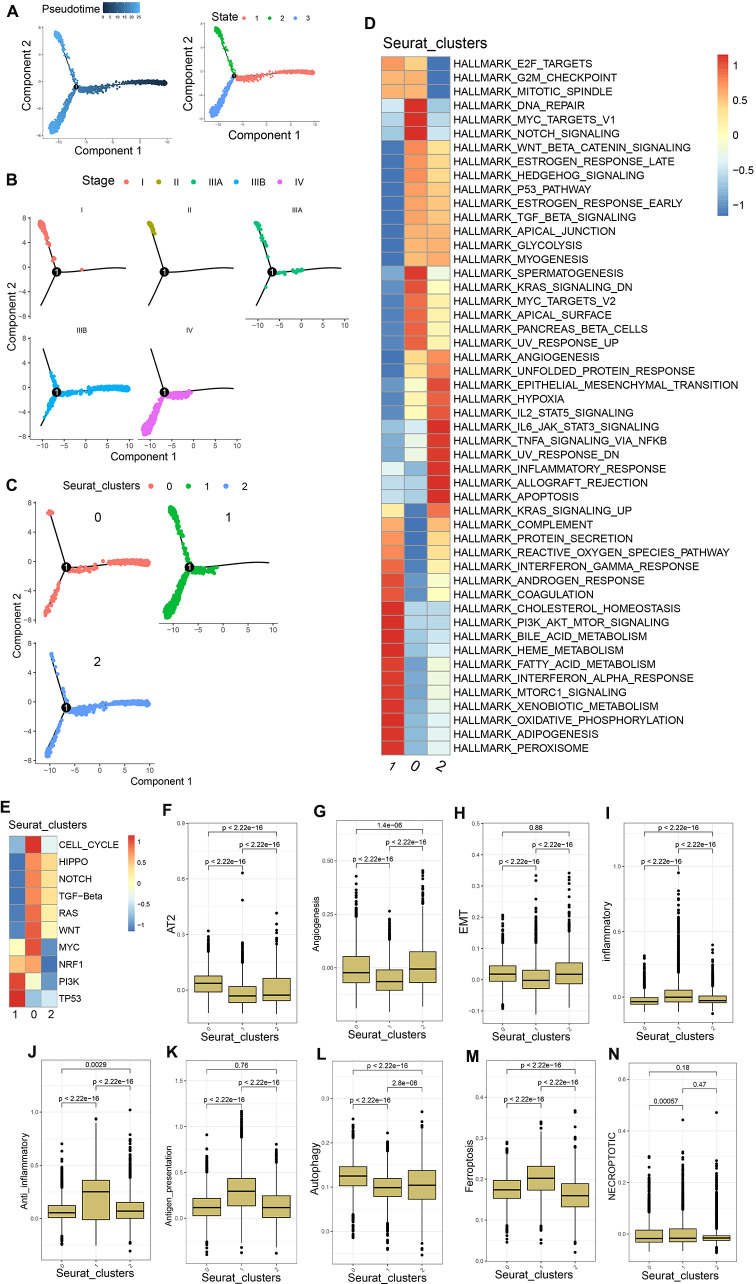
PVTT cell characteristics. **(A)** Pseudotime analyses were conducted for the mixed population of PT and PVTT cells. **(B)** HCC cell developmental trajectories were assessed for different pathological stages. **(C)** HCC cell developmental trajectories were assessed for different clusters. **(D)** GSVA enrichment analyses were performed for cells in clusters 0, 1, and 2. **(E)** GSVA analyses of pathways associated with tumor activation were conducted for cells in clusters 0, 1, and 2. **(F-N)** AT2, angiogenesis, EMT, inflammatory response, anti-inflammatory response, antigen presentation, autophagy, ferroptosis, and necroptosis scores were generated for cells in clusters 0, 1, and 2.

Pseudotime analysis trajectories revealed three different HCC cell developmental trajectories numbers states 1-3 (**Figure 3A**). The HCC cells from patients with stage I-IIIA disease underwent differentiation from state 1 to state 2, whereas those from patients with stage IIIB disease underwent differentiation from state 1 to states 2 and 3, and those from stage IV patients underwent differentiation from state 1 to state 3 (**Figure 3B**). The differentiation trajectories of HCC cells also varied as a function of clinical malignancy, with cells in clusters 1 and 2 exhibiting trajectories similar to those of pathological stage IIIB HCC cells, while cells in cluster 0 exhibited trajectories consistent with those of pathological stage IV HCC (**Figure 3C**). These findings suggested that PVTT cells exhibit greater malignancy, with developmental trajectories significantly distinct from those of HCC cells in earlier stages of pathological development.

In GSVA enrichment analyses exploring differences in the biological characteristics of cells in the three established clusters, cluster 0 exhibited enrichment for the DNA repair, Myc targets V1, E2F targets, G2M checkpoint, mitotic spindle, and other pathways. Cluster 1 exhibited enrichment for the coagulation, complement, interferon-gamma response, bile acid metabolism, and other pathways associated with tumor matrix activation and inflammatory activity. Cluster 2 exhibited enrichment for the IL6 JAK STAT3 signaling, TNFA signaling via NFkB, allograft rejection, IL2 STAT5 signaling, and other immunosuppression and immune inflammation-related pathways (**Figure 3D**). GSVA analyses of 10 tumor activation-related pathways further demonstrated that cells from cluster 0 were enriched in 8 pathways including the cell cycle, Wnt, Hippo, and TGF-β pathways, whereas cluster 1 cells were enriched in the PI3K and TP53 pathways, and cluster 2 cells were enriched in the Wnt, Hippo, Notch, and TEG-β pathways, albeit with weak expression (**Figure 3E**). The biological process-associated gene set was used to score cells in these three clusters, revealing that cluster 0 cells presented with higher AT2, EMT, and angiogenesis scores relative to cluster 1 cells, together with lower inflammatory response, anti-inflammatory response, and antigen presentation scores. With respect to types of cell death, the autophagy scores in cluster 0 were higher than those in cluster 1, whereas cluster 1 cells exhibited higher necroptosis and ferroptosis scores relative to cluster 0 (**Figures 3F–N**). These data together suggest that PVTT cells are primarily represented by cells in cluster 0, which exhibited characteristics including tumor cell proliferation, the activation of the tumor stroma, and lower levels of immune infiltration consistent with an immune desert and immune rejection. In contrast, PT samples were represented by the cells in cluster 1, which exhibited evidence of inflammatory activity The mixed PT and PVTT cells present in cluster 2 presented with a phenotype consistent with f immune rejection.

### Core PVTT gene selection and characterization

Using the R FindAllMarkers function, the marker genes associated with each of these established cell clusters were identified (**Supplementary Figure 1A**), yielding 6,364 marker genes characteristic of the cells in cluster 0. Next, bulk RNA-seq datasets from the TCGA and ICGC databases were downloaded, and comparisons of the HCC and normal liver tissue samples in these datasets were used to identify DEGs, after which DEGs significantly associated with patient OS were screened with univariate Cox regression analyses.

**Figure 4:**
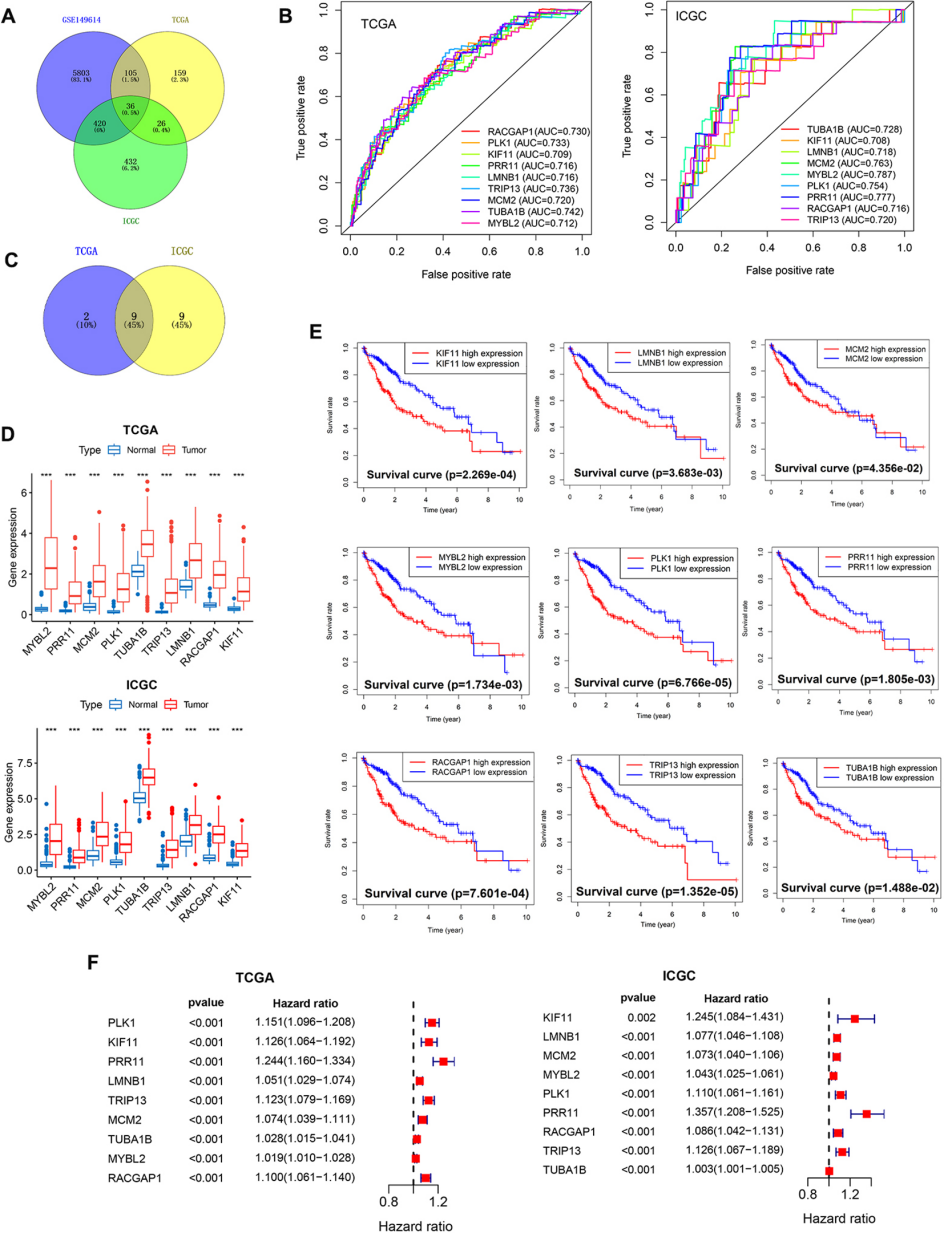
Core PVTT-associated gene screening and characterization. **(A)** 36 candidate genes characteristic of PVTT were identified through screening. **(B)** AUC values were calculated for the 9 core genes to assess their prognostic performance in HCC patients. **(C)** Screening of the overlap between the 9 core genes and PVTT-associated characteristic genes. **(D)** Analyses of the differential expression of the 9 core genes in HCC and normal liver tissue samples from the TCGA and ICGC datasets. **(E)** The relationship between the expression levels of 9 genes and HCC patient survival for individuals in the TCGA cohort. **(F)** Univariate Cox regression analyses of the expression of 9 genes and the survival of patients with HCC in the TCGA and ICGC cohorts. ***P<0.001.

Ultimately, 36 candidate genes were obtained based on the overlap between the 6,365 cluster 0 marker genes and those genes significantly related to the OS of patients in the TCGA and ICGC data sets (**Figure 4A**). In order to facilitate subsequent model development, ROC curves for each of these 36 candidate genes were used to assess their prognostic performance in the TCGA and ICGC datasets. Those genes with ROC values > 0.7 in these datasets were then selected, yielding 9 overlapping genes defined as core genes of interest for further analyses (**Figures 4B, C**). These genes included MYBL2, PRR11, MCM2, PLK1, TUBA1B, TRIP13, LMNB1, RACGAP1, and KIF11. All 9 of these genes were expressed at higher levels in HCC tissue samples relative to normal liver tissue in the TCGA and ICGC cohorts (**Figure 4D**), and the elevated expression of all 9 genes was related to shorter HCC patient OS (**Figure 4E**; **Supplementary Figure 1B**). Univariate Cox regression analyses were performed to assess the expression of these genes in the TCGA and ICGC datasets and their relationship with patient survival status, confirming that high levels of all 9 of these genes were independently predictive of HCC patient OS (**Figure 4F**).

**Figure 5:**
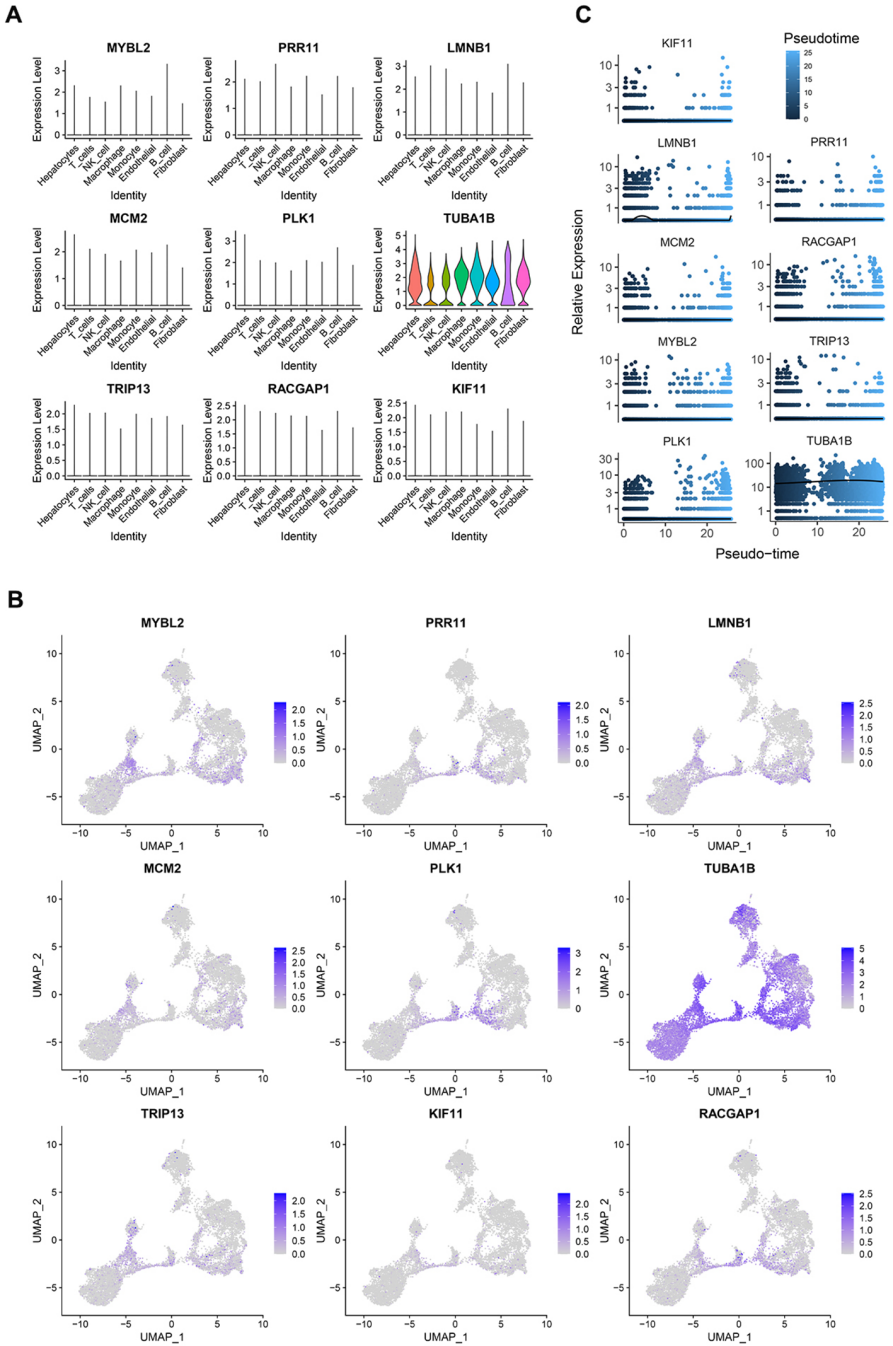
PVTT core gene expression profiles. **(A)** The expression of the 9 core genes across different cell types in single-cell analyses. **(B)** The distribution of the 9 core genes in cells from clusters 0 and 1. **(C)** The results of a pseudotime analysis of these 9 core genes.

These 9 genes were expressed across all 8 cell types included in these single-cell analyses (**Figure 5A**). In the extracted PVTT samples, the PRR11, PLK1, and RACGAP1 core genes were primarily found at the junction between the cells in clusters 0 and 1, with this area essentially overlapping with the distribution of cells in the G2M phase of the cell cycle (**Figure 5B**). Pseudotime analyses of these 9 genes revealed slight fluctuations in LMNB1 expression early during HCC cell development, whereas the remaining 8 genes presented relatively stable patterns of expression over the course of HCC development (**Figure 5C**). This suggests that the core PVTT-related genes identified herein exhibit biological characteristics consistent with rapid HCC cell proliferation and division.

### Core gene functional enrichment, protein interaction, and genetic variation analyses

**Figure 6:**
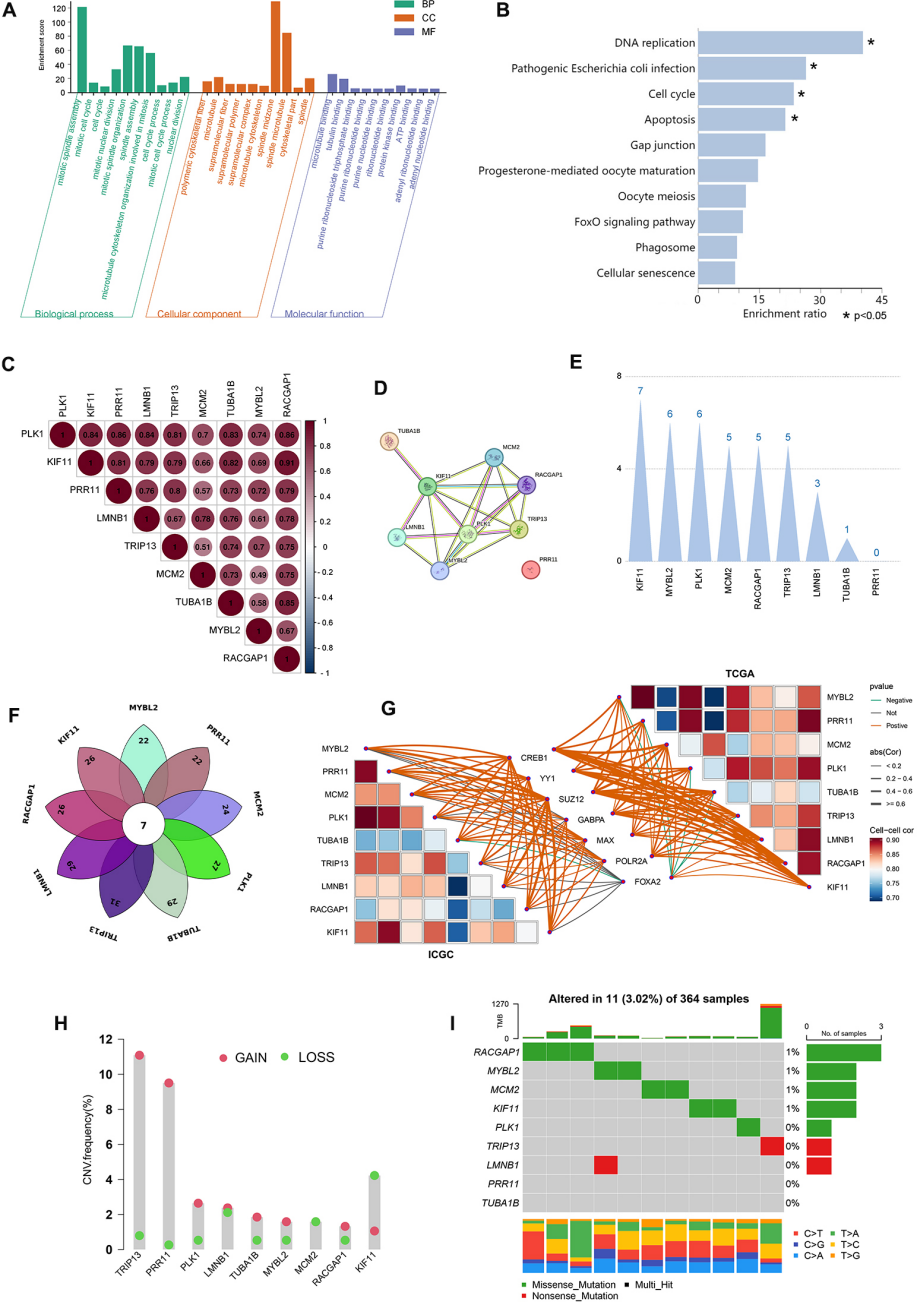
Core gene functional enrichment, protein interaction, and genetic variation analyses. **(A, B)** The 9 core genes were subjected to GO and KEGG enrichment analyses. **(C)** Correlation analyses were performed assessing the expression of the 9 core genes. **(D)** Protein interactions were assessed for the 9 core genes. **(E)** Weighting analyses of protein interactions were conducted among these 9 core genes. **(F)** The transcription factors that regulate the 9 core genes are intersected. **(G)** Correlation analysis of 7 common transcription factors with 9 core genes in TCGA and ICGC datasets. **(H, I)** These 9 core genes were assessed for copy number variations and somatic mutations.

Next, these 9 core genes were subjected to GO and KEGG enrichment analyses, revealing that they were primarily enriched in proliferation-related biological processes including the cell cycle, mitotic cell cycle, and mitotic spindle assembly. They were also enriched for cellular components including microtubules, polymeric cytoskeletal fibers, and supramolecular fibers. They were further enriched in molecular functions including tubulin binding, microtubuline binding, and purine ribonucleoside triphosphate binding (**Figure 6A**). Lastly, they were enriched in the apoptosis, cell cycle, DNA replication, and pathogenic Escherichia coli infection KEGG pathways (**Figure 6B**).

Correlation analyses of the expression of these 9 core genes revealed that their expression patterns were significantly related to one another, with correlation coefficients from 0.7-0.86 (P<0.001) (**Figure 6C**). All of the proteins encoded by these core genes other than PRR11 were predicted to interact with one another (**Figure 6D**). Specifically, KIF11 presented with 7 interacting relationships, exhibiting the most weight, followed by RACGAP1 with 5 interactions. In contrast, PRR11 exhibited the least weight as it was not predicted to interact with any of these other proteins (**Figure 6E**). How KIF11, RACGAP1, and RPR11 impacted the expression of other genes was assessed, revealing that changes in the expression of any of these three genes would result in significant, comparable changes in the expression of the remaining 8 genes (**Supplementary Figure 1D**). These evidences suggested that the 9 core genes may share common transcriptional regulators, and the changes in the expression of the transcriptional regulators can simultaneously affect the expression of the 9 core genes, resulting in obvious biological effects. Through the online website hTFtarget (wchscu.cn), the transcription factors of 9 core genes in liver tissues were retrieved, and the obtained data were overlapped to obtain a total of 7 intersection genes (**Figure 6F**). The 7 transcription factors are FOXA2, CREB1, GABPA, MAX, POLR2A, YY1 and SUZ12. Correlation analysis of 7 transcription factors and 9 core genes revealed that the top 3 transcription factors with the highest correlation with 9 core gene were CREB1, YY1 and SUZ12 in TCGA and ICGC databases (**Figure 6G**). In addition, there was also protein interaction among 7 transcription factors ( **Supplementary Figure 2A**), and the top 3 transcription factors with the closest interaction were YY1, CREB1 and POLR2A (**Supplementary Figure 2A**).

Copy number variation (CNV) and somatic mutation incidence in these 9 core PVTT-associated genes were next analyzed. High CNV frequencies were observed in these genes, with MYBL2, PLK1, TUBA1B, TRIP13, LMNB1, RACGAP1, and PRR11 generally exhibiting copy number amplifications whereas KIF11 and MCM2 more frequently exhibited copy number deletions (**Figure 6H**). To determine whether these genetic variants impacted the expression of these 9 core genes in patients with PVTT, their expression patterns in HCC and normal tissue samples were examined, revealing that the alterations of CNV could be the prominent factors resulting in perturbations on the PVTT core genes expression. With respect to somatic mutations across 364 samples, just 11 samples presented with mutations in these 9 core genes (3.02%), with the highest mutation frequencies being evident for RACGAP1, MYBL2, and MCM2, while missense mutations were the most common type of mutation. No somatic mutations were observed in PR11 or TUBA1B across the analyzed samples (**Figure 6I**). These results emphasize the highly heterogeneous nature of the genomic and expression landscapes characterizing these core PVTT-related genes, suggesting that their dysregulated expression likely plays a central role in the onset and progression of PVTT.

### The relationships between core gene expression, patient prognosis, and immune infiltration

**Figure 7:**
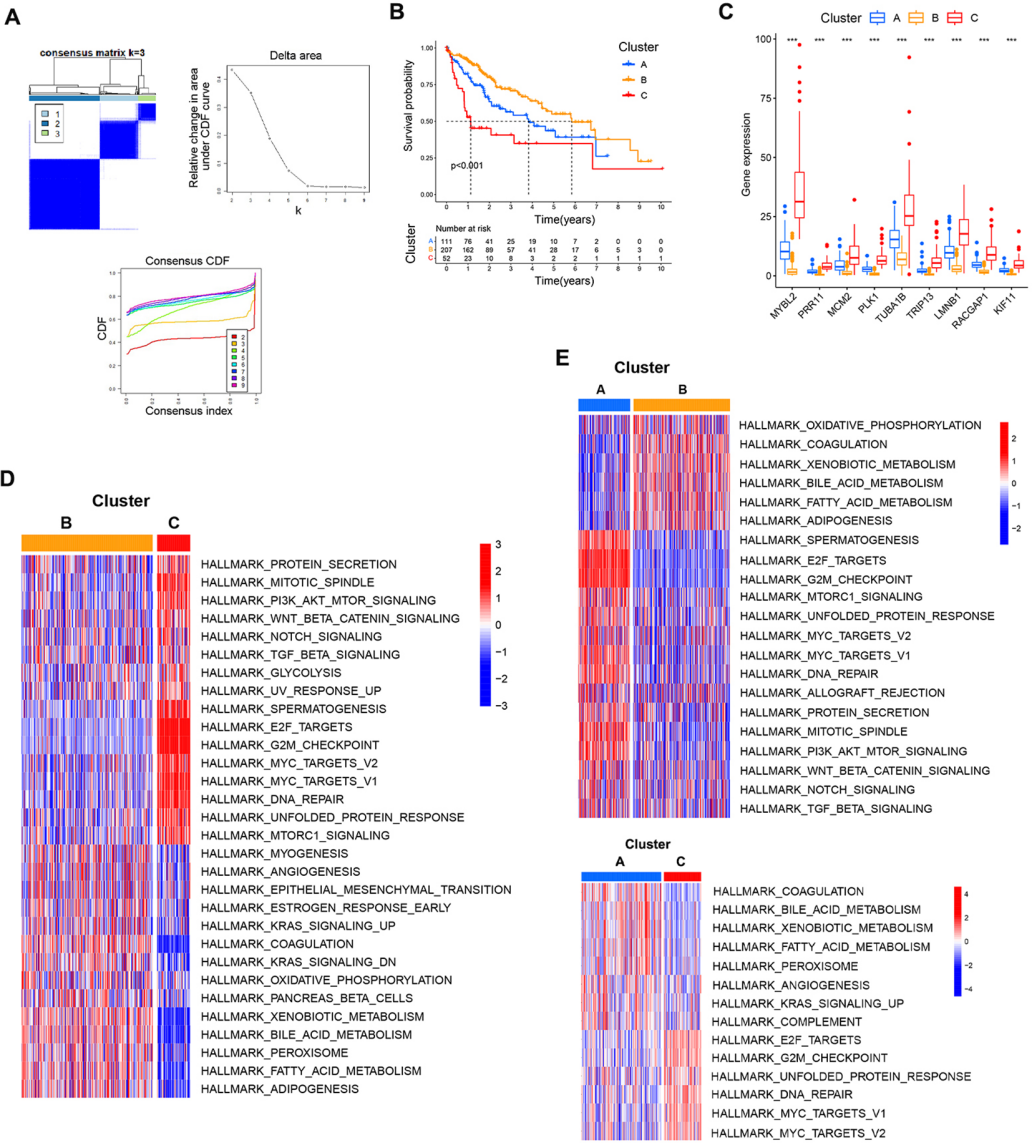
Associations between core gene expression and clustering patterns. **(A)** Consensus matrices for the TCGA dataset at k values from 2-4. **(B)** The effect of these three patterns of clustering on patient prognostic outcomes. **(C)** Analyses of differences in core gene expression for each clustering pattern. **(D, E)** Pairwise GSVA enrichment analyses for the three established patterns of clustering. ***P<0.001.

After extracting the expression matrix corresponding to these 9 core genes from the TCGA database, HCC patients were classified according to the expression patterns for these genes using the R ConsensusClusterPlus package through an unsupervised approach that ultimately assigned 111, 207, and 52 cases to clusters A, B, and C, respectively (**Figure 7A**). Prognostic analyses of these clusters demonstrated that the cluster B expression pattern was associated with a pronounced survival advantage, whereas the cluster C pattern was linked to markedly worse survival (**Figure 7B**). The relationships between the expression levels for these 9 core genes and their clustering patterns revealed that their expression levels rose progressively from cluster B to cluster A to cluster C (**Figure 7C**). These findings suggested that changes in the expression of core genes will result in altered clustering patterns that may be relevant to patient prognosis.

To better understand biological behaviors associated with these clustering patterns, GSVA enrichment analyses were conducted. Cluster B presented with the pronounced enrichment of pathways associated with matrix activation including the EMT, angiogenesis, and bile acid metabolism pathways, as well as inflammatory pathways including the KRAS signaling and coagulation pathways. Cluster C presented with the marked enrichment of oncogenic activation pathways including the mitotic spindle, DNA repair, G2M checkpoint, and E2F target pathways. Cluster A exhibited clear inflammatory activation pathway enrichment and the enrichment of matrix activation pathways including the complement, angiogenesis, bile acid metabolism, and KRAS signaling pathways (**Figures 7D, E**).

**Figure 8:**
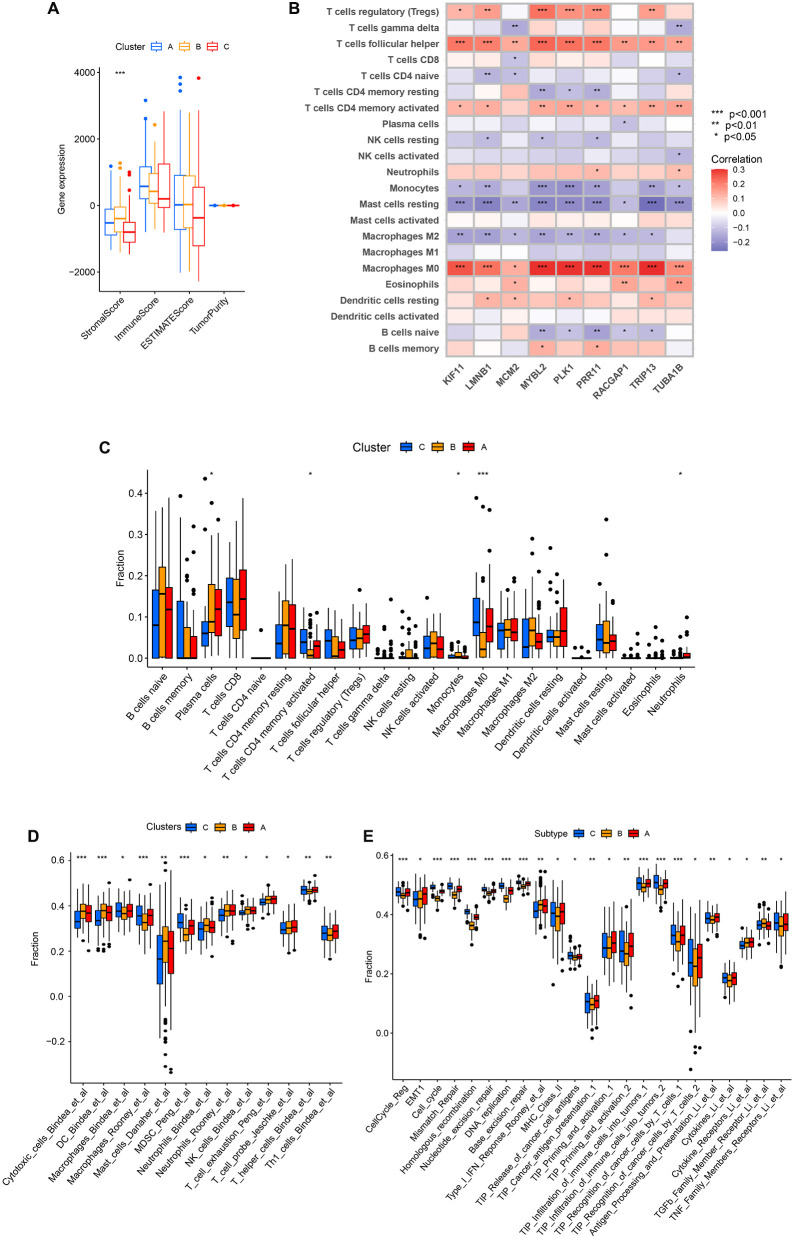
The characteristics of TME cell infiltration for different clustering patterns. **(A)** TME scores for the indicated clustering patterns. **(B)** Correlations between the expression of the 9 core genes and immune cell infiltration. **(C)** TME-infiltrating cell abundance for each of the indicated clustering patterns. **(D)** The IOBR package was used to assess the abundance of TME-infiltrating cells across the indicated clustering patterns. **(E)** Differences in tumor stroma scores and the tumor-immune cycle were assessed across the indicated clustering patterns. *P<0.05, **P<0.01, ***P<0.001.

Differences in immune cell infiltration across these three patterns of clustering were next probed with the ESTIMATE algorithm, revealing that only StromalScore values differed significantly among these three clusters, with clusters B and C respectively exhibiting the highest and lowest StromalScore values (**Figure 8A**). This suggests that cluster B was associated with significant activation of the tumor stroma. Using the CIBERSORT method, immune cell infiltration was next analyzed across these three clustering patterns. Through this approach, cluster B was found to exhibit greater monocyte infiltration, whereas cluster A presented with more pronounced neutrophil and plasma cell infiltration, and cluster C exhibited greater infiltration by M0 macrophages and activated memory CD4 T cells (**Figure 8C**). Given that these three clustering patterns were closely associated with the expression patterns for these 9 core genes, correlations between core gene expression and immune cell infiltration were assessed, revealing that the expression of these 9 core genes was positively correlated with infiltration by Tregs, Tfh cells, activated memory CD4 T cells, and M0 macrophages, whereas it was negatively correlated with infiltration by monocytes, M2 macrophages, and resting mast cells (**Figure 8B**). This suggests that the enhanced expression of these 9 core genes was related to reduced infiltration by immunoinflammatory effectors cells together with enhanced infiltration by immunosuppressive cells and the decrease of immunoinflammatory cells. Based on these results, clusters A and B were classified as exhibiting an immune-excluded phenotype characterized by stromal activation and infiltration by innate immune cells, while cluster C was classified as exhibiting an immune desert phenotype consistent with immune suppression.

Specific correlations between infiltration by immune cells and patterns of clustering were further assessed with the IOBR package. Through this approach, cluster B was found to exhibit significantly greater enrichment in infiltration by innate immune cell populations including cytotoxic cells, dendritic cells, mast cells, neutrophils, neutrophils, and NK cells. In contrast, cluster A presented with significantly greater enrichment of exhausted T cells, probe T cells, and Th1 cells, while cluster C presented with more enriched infiltration by macrophages, MDSCs, and T helper cells (**Figure 8D**). Tumor stroma scores were also compared across these three clustering patterns, revealing that cluster B was positively associated with certain signals of tumor stroma activation including the type I IFN response, cytokine receptors, and TGF-β family member receptors. Cluster C, in contrast, exhibited positive correlations with certain signals associated with oncogenic activation including the cell cycle, mismatch repair, homologous recombination, and DNA replication (**Figure 8E**). These results are consistent with the previous analysis, indicating that cluster A and cluster B were classified as exhibiting an immune-excluded phenotype, and cluster C cluster was classified as exhibiting an immune desert phenotype.

Analyses of differences in the tumor-immune cycle across these three clusters revealed that cluster A was positively associated with TIP Cancer antigen presentation, TIP Priming and activation, TIP Recognition of cancer cells by T cells, Antigen Processing and Presentation, Cytokines, and Cytokine Receptors. This suggests that cluster A samples are more likely to undergo immune-mediated recognition and the consequent engagement of an antitumor immune response (**Figure 8E**).

### Development of a core gene-based prognostic model

To better define the features of TME cell infiltration in HCC in the presence or absence of PVTT, multivariate Cox regression analyses were used to develop a prognostic model incorporating three of the nine core genes (PRR11, KIF11, and RACGAP1) with the following formula: risk score = KIF11*-0.194635376203241+PRR11*0.232461703663777+RACGAP1* 0.112717648336317.

**Figure 9:**
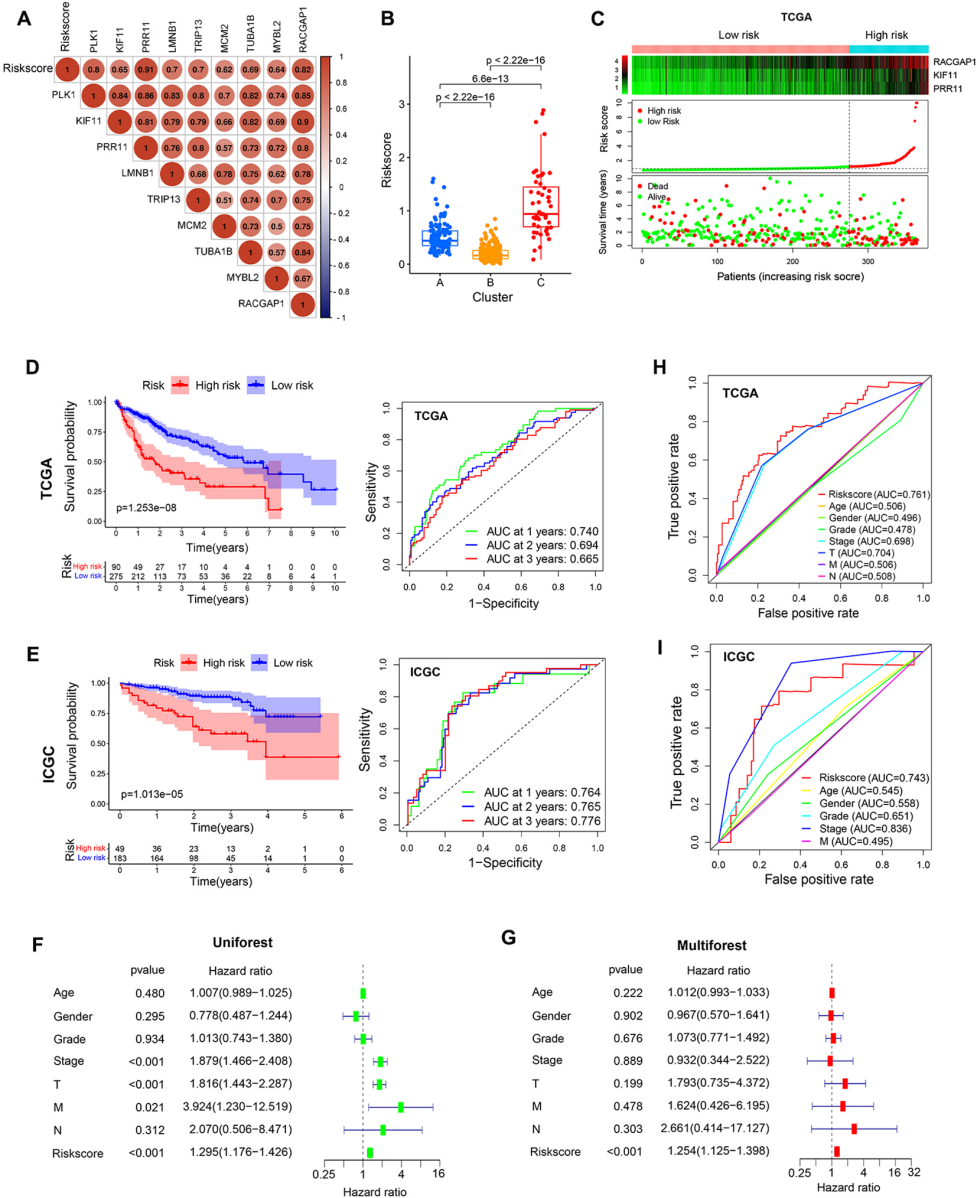
Prognostic model development and evaluation. **(A)** Analysis of correlations between risk scores and core genes. **(B)** Analyses of differences in risk scores in different clusters. **(C)** The survival status of patients in different risk groups in the TCGA cohort. **(D, E)** The association between risk scores and prognostic outcomes for TCGA and ICGC patients. **(F, G)** Univariate and multivariate Cox analyses of patient risk score values and clinical characteristics. **(H, I)** AUC values for risk scores and other clinicopathological characteristics as predictors of the prognostic outcomes for TCGA and ICGC patients.

Correlation analyses of the relationships between core genes or clustering patterns and risk scores indicated that the expression of the 9 core genes was positively associated with risk score values (**Figure 9A**). Moreover, significantly higher risk scores were observed in cluster C relative to the other clusters, whereas cluster B exhibited lower risk scores (**Figure 9B**). Median risk scores were then used to separate patients into low-risk (LR) and high-risk (HR) groups to compare prognostic outcomes for these two sets of patients (**Figure 9C**). Kaplan-Meier curves indicated that patients in the LR group presented with a significant survival advantage. ROC curves were further used to predict patient 1-, 2-, and 3-year OS, yielding respective AUC values of 0.74, 0.694, and 0.665 in the TCGA-LIHC cohort (**Figure 9D**). Consistently, the LR group exhibited clear survival advantages in the ICGC-LIHC cohort (**Supplementary Figure 1C**), with respective AUCs of 0.764, 0.756, and 0.776 (**Figure 9E**). In univariate and multivariate analyses of risk scores and patient clinicopathological characteristics including age, gender, tumor grade, clinical stage, and survival status, risk scores remained significantly independently associated with HCC patients OS (**Figures 9F, G**). Risk scores also exhibited AUC values (0.761) superior to those of tumor stage or grade in terms of predictive value in the TCGA cohort, suggesting that this risk scoring can be used to more effectively predict the prognostic outcomes of patients with HCC (**Figure 9H**). The model also offered value for the prognostic assessment of the patients included in the ICGC dataset (**Figure 9I**).

**Figure 10:**
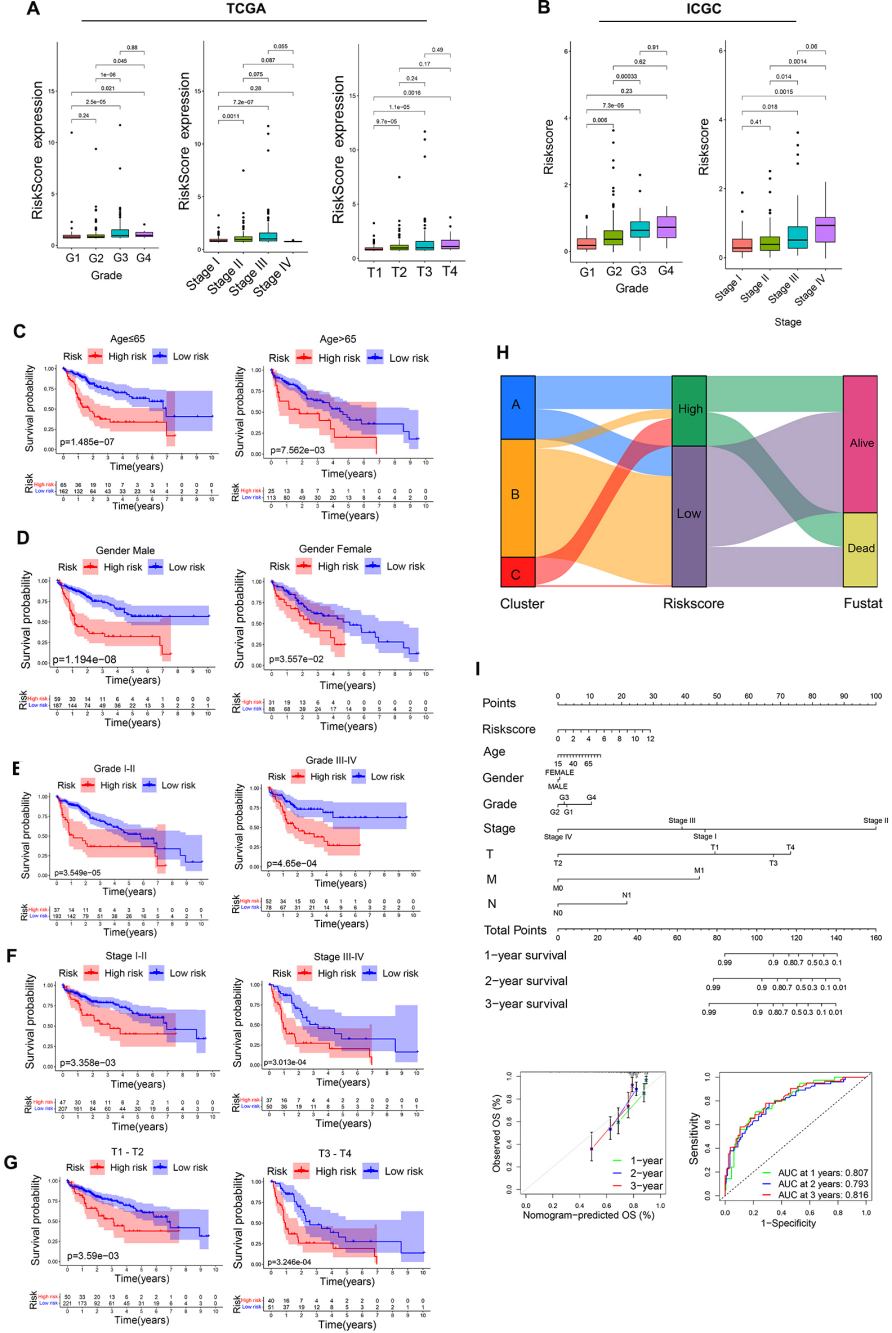
Assessment of prognostic model predictive performance. **(A)** The associations between risk scores and pathological grade, pathological stage, and T stage in the TCGA cohorts. **(B)** The associations between risk scores and pathological grade, pathological stage in ICGC cohorts. **(C-G)** Risk score distributions for different patient subsets within the TCGA cohort. **(H)** A Sankey diagram corresponding to clustering patterns and survival outcomes. **(I)** Nomogram construction.

Analyses of intragroup differences and clinical characteristics revealed significant differences in risk scores among patients with different pathological grades, pathological stages, and T stages in both the TCGA (**Figure 10A**) and ICGC cohorts (**Figure 10B**). Risk scores rose with increasing pathological grade, pathological stage, and T stage. When HCC patients were stratified into subgroups based on their clinical characteristics, risk score values were found to offer utility for the identification of patient age (≤65 vs. >65 years) (**Figure 10C**), gender (female vs. male) (**Figure 10D**), pathological grade (GI-II vs. GIII-IV) (**Figure 10E**), pathological stage (SI-II vs. SIII-IV) (**Figure 10F**), and T stage (T1-2 vs. T3-4) (**Figure 10G**). The same was true in the ICGC validation cohort (**Supplementary Figure 2B**), suggesting that this model offers a high degree of sensitivity for the prediction of prognostic outcomes for patients with HCC.

Associations between risk score values, clustering patterns, and patient prognostic outcomes were also assessed, revealing that the LR group was primarily comprised of cluster B and small amounts of cluster A, whereas most of clusters A and C were included in the HR group (**Figure 10H**). With respect to prognostic outcomes, most patients from cluster C and some from cluster B were dead, while most patients in cluster A remained alive despite their high risk score values. These findings, together with the tumor-immune cycle results for these different clusters, may suggest that tumors from patients in cluster A can be more readily recognized by the immune system, activating the antitumor immune response. To better aid efforts to evaluate patient prognostic outcomes based on these parameters, a nomogram was developed incorporating risk score, patient age, sex, pathological stage, pathological grade, and other variables (**Figure 10I**). Multivariate ROC analyses indicated that the developed prognostic variable was able to effectively predict HCC patient 1-year (AUC=0.807), 2-year (AUC=0.793), and 3-year (AUC=0.816) OS, with curve fitting exhibiting good reliability (**Figure 10I**).

### Risk score, immunotherapy, and drug sensitivity analyses

**Figure 11:**
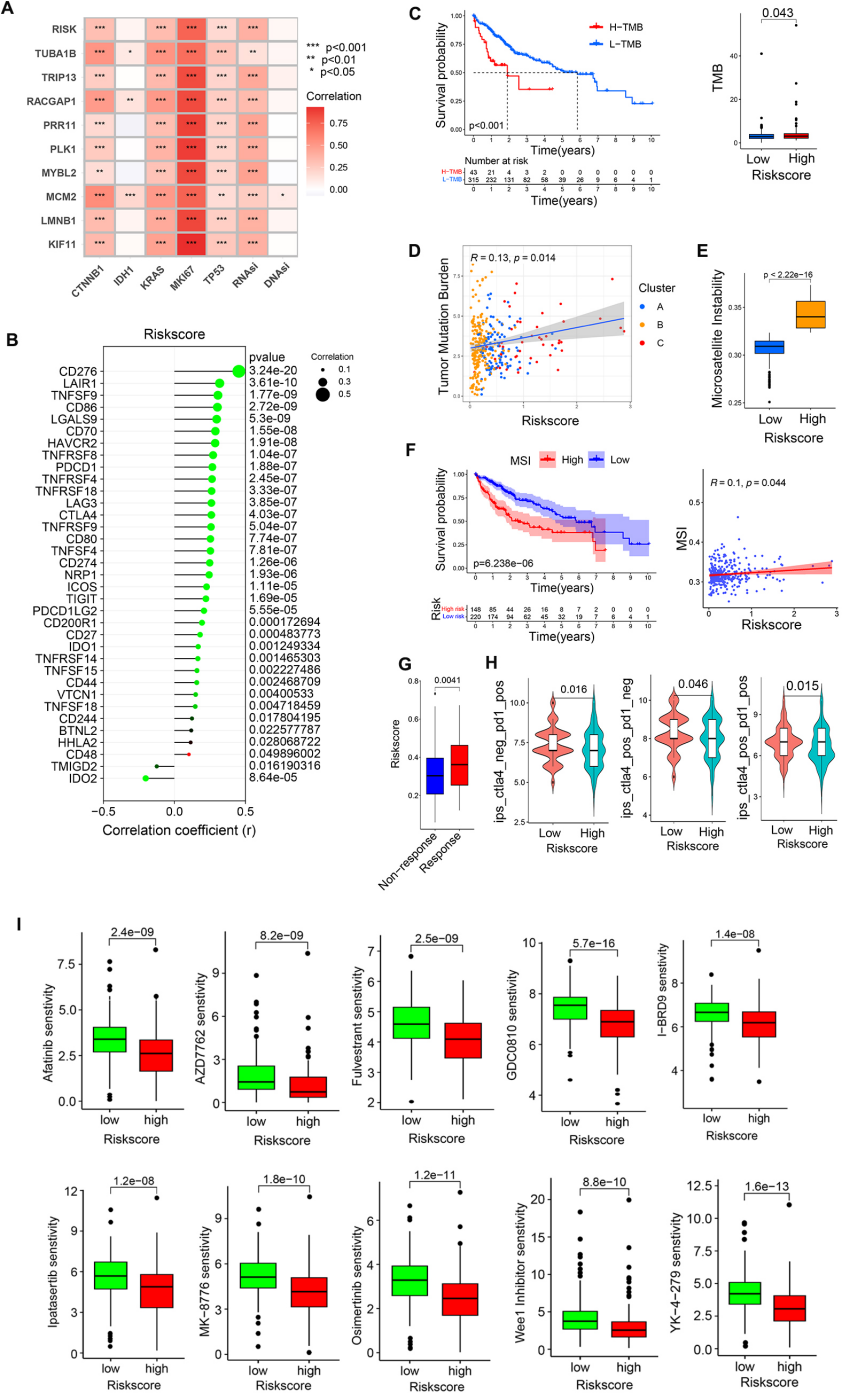
The relationship between risk scores and patient immunotherapy or drug sensitivity. **(A)** Correlation analyses of the relationships between pro-tumor factors, stem cell indices, and risk score values. **(B)** Correlation analyses of the relationship between risk scores and immune checkpoints. **(C, D)** Correlation analyses of the relationship between TMB and risk scores. **(E, F)** Correlation analyses of the relationship between MSI and risk scores. **(G)** Analysis of ICB responses for patients with low and high risk scores. **(H)** Sensitivity analyses of patients with low and high risk scores to chemotherapeutic agents in the GDSC2 database. **(I)** Screening chemotherapy drugs with potential therapeutic value for high-risk patients. *P<0.05, **P<0.01, ***P<0.001.

Correlations between risk scores and both pro-tumor factors and stem cell indices were next assessed, revealing that risk scores and the 9 core genes were all strongly positively correlated with MIK67, CTNNB1, KRAS, TP53, and RNAsi, whereas they were not correlated with IDH1 or DNAsi (**Figure 11A**). This is consistent with higher levels of pro-tumor factor activity in the HR group. Correlations between risk scores and immune checkpoints also supported an association between high risk scores and immune checkpoint expression (**Figure 11B**), consistent with higher levels of checkpoint-mediated suppression in the HR group.

In tumor mutational burden (TMB) analyses, a higher level of TMB in HCC patients was associated with significant survival disadvantages, with HR patients exhibiting a higher TMB (**Figure 11C**), and a positive correlation between TMB and risk scores (**Figure 11D**). HCC patients exhibiting high levels of microsatellite instability (MSI) also presented with a clear survival disadvantage relative to those with low MSI scores (**Figure 11F**). High-risk patients also presented with higher MSI scores (**Figure 11E**), and there was a positive correlation between risk scores and MSI scores (**Figure 11F**).

To clarify the responses of low- and high-risk patients to immunotherapy, extant immunotherapy cohort data from The Cancer Immunome Atlas (TCIA) cohort study (**Figure 11H**) and the Urothelial Cancer Treatment Study (IMvigor) (**Figure 11G**) were utilized. This approach revealed that high-risk patients were more likely to respond to PD-1 or CTLA-4 targeting therapies when provided alone or in combination with one another. In contrast, the Melanoma Treatment study (GSE78220), and Renal Cancer Treatment Study (GSE67501) datasets revealed no apparent positive treatment responses for patients in the low- or high-risk groups (**Supplementary Figure 2C**).

Next, GDSC (Genomics of Drug Sensitivity in Cancer) data were leveraged to screen for chemotherapeutic drugs that may offer value for high-risk patient treatment. The 10 drugs with the highest levels of sensitivity were Afatinib, AZD7762, Fulvestrant, GDC0810, I-BRD9, Ipatasertib, MK-8776, Osimertinib, Wee1 Inhibitor, YK-4-279 (**Figure 11I**).

### PRR11, KIF11, RACGAP1, YY1 and CREB1 is highly expressed in HCC tissue

**Figure 12:**
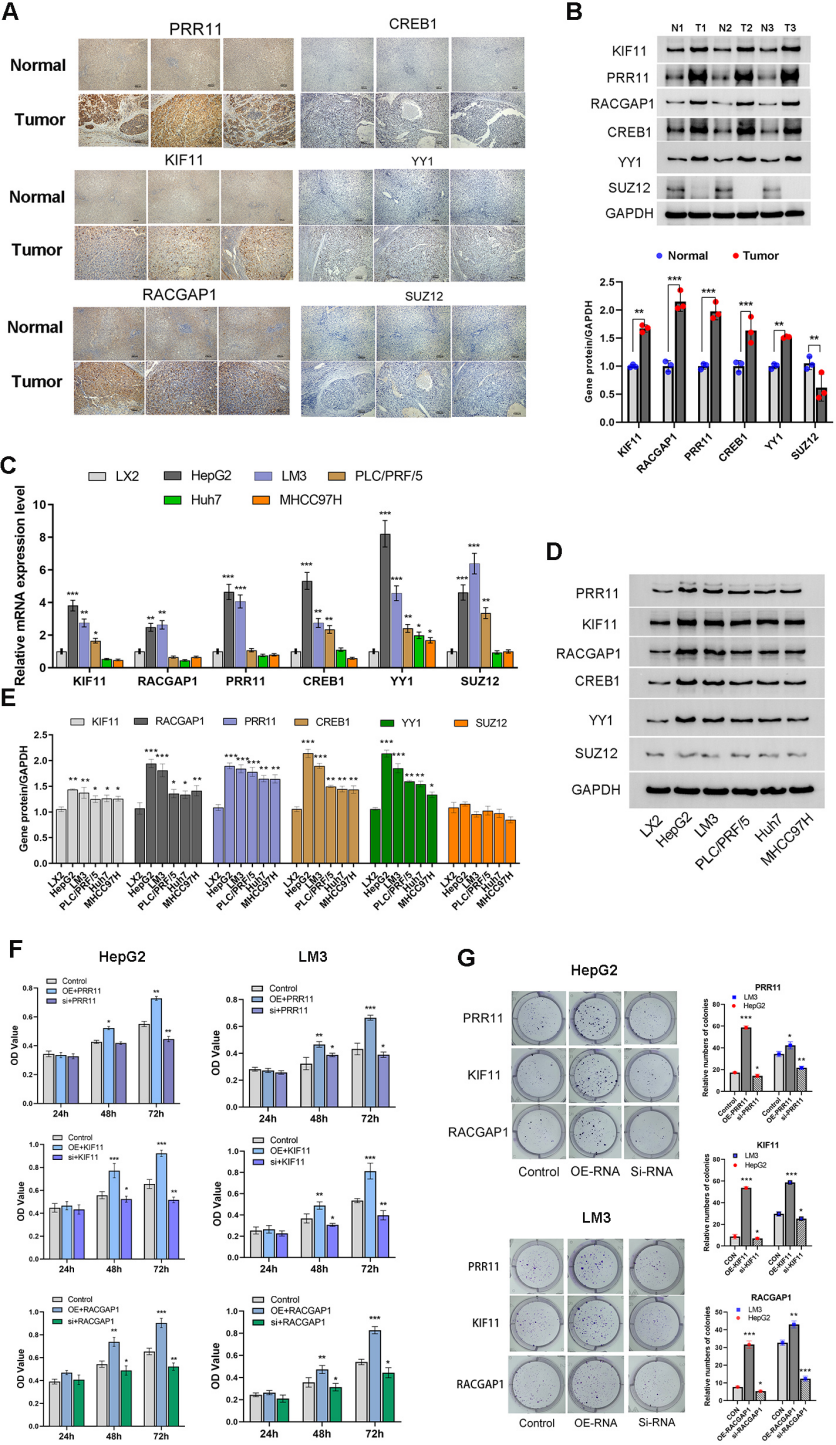
PRR11, KIF11, RACGAP1, YY1 and CREB1 is highly expressed in HCC tissue and HCC cell lines. **(A, B)** Immunohistochemical staining and Western blotting analysis of PRR11, KIF11, RACGAP1, YY1, CREB1 and SUZ12 in HCC tissues and para-tumor tissues. **(C-E)** The mRNA and protein expression of PRR11, KIF11, RACGAP1, YY1, CREB1 and SUZ12 in Cells line (LX2, HepG2, HCC-LM3, Huh-7, PLC/PRF/5 and MHCC97H). **(F, G)** Cell viability and colony formation assay shows the effect of PRR11, KIF11, RACGAP1 expression levels on cell viability and colony formation abilities of HepG2 and HCC-LM3 cells. *P<0.05, **P<0.01, ***P<0.001.

To validate the bioinformatics findings, HCC tissues and para-tumor tissues were collected. Immunohistochemical staining and Western blotting were used to observe the expression of risk model factors PRR11, KIF11, RACGAP1 as well as the potential common transcription factors YY1, CREB1 and SUZ12 in HCC tissues and para-tumor tissues (**Figures 12A, B**). IHC score indicated that PRR11, KIF11, RACGAP1, YY1, CREB1 expression was significantly increased in HCC tissues compared to adjacent para-tumor tissues (**Supplementary Figure 2D**). However, the protein expression of SUZ12 in HCC tissues was lower than that in adjacent para-tumor tissues (**Figure 12B**).

### High expression of PRR11, KIF11, RACGAP1, YY1 and CREB1 promotes tumorigenic properties of HCC cells

To investigate the potential roles of PRR11, KIF11, RACGAP1, YY1, CREB1 and SUZ12 in the development of HCC. Human Hepatic Stellate Cells line (LX2) and five HCC cell lines (HepG2, HCC-LM3, Huh-7, PLC/PRF/5, MHCC97H) were used to detect the mRNA and protein expression of PRR11, KIF11, RACGAP1, YY1, CREB1 and SUZ12 (**Figures 12C–E**). The results indicated that there was no statistical difference in protein expression of SUZ12 between HCC cell lines and LX2 cell line, and the mRNA and protein expressions of PRR11, KIF11, RACGAP1, YY1, CREB1 were significantly higher than those of LX-2 cell line, especially HepG2 and HCC-LM3 cell lines (**Figures 12D, E**). Therefore, HepG2 and HCC-LM3 cell lines with highest expression of model factors were selected for subsequent experiments.

Transient transfection was used to detect the effect of model factors on the phenotype of HCC cells. Overexpression of PRR11, KIF11 and RACGAP1 was found to promote cell viability of HepG2 and HCC-LM3 cell lines at 48 h and 72h. The cell viability of silencing group of PRR11, KIF11 and RACGAP1 was shown to be significantly lower than that of control group at 48 h and 72h. PRR11, KIF11 and RACGAP1 silencing was found to reduce the number of clones of HepG2 and HCC-LM3 cell lines compared to those of the control groups (**Figure 12F**). And overexpression of PRR11, KIF11 and RACGAP1 was found to increase the number of clones of HepG2 and HCC-LM3 cell lines compared to those of the control groups (**Figure 12G**).

**Figure 13:**
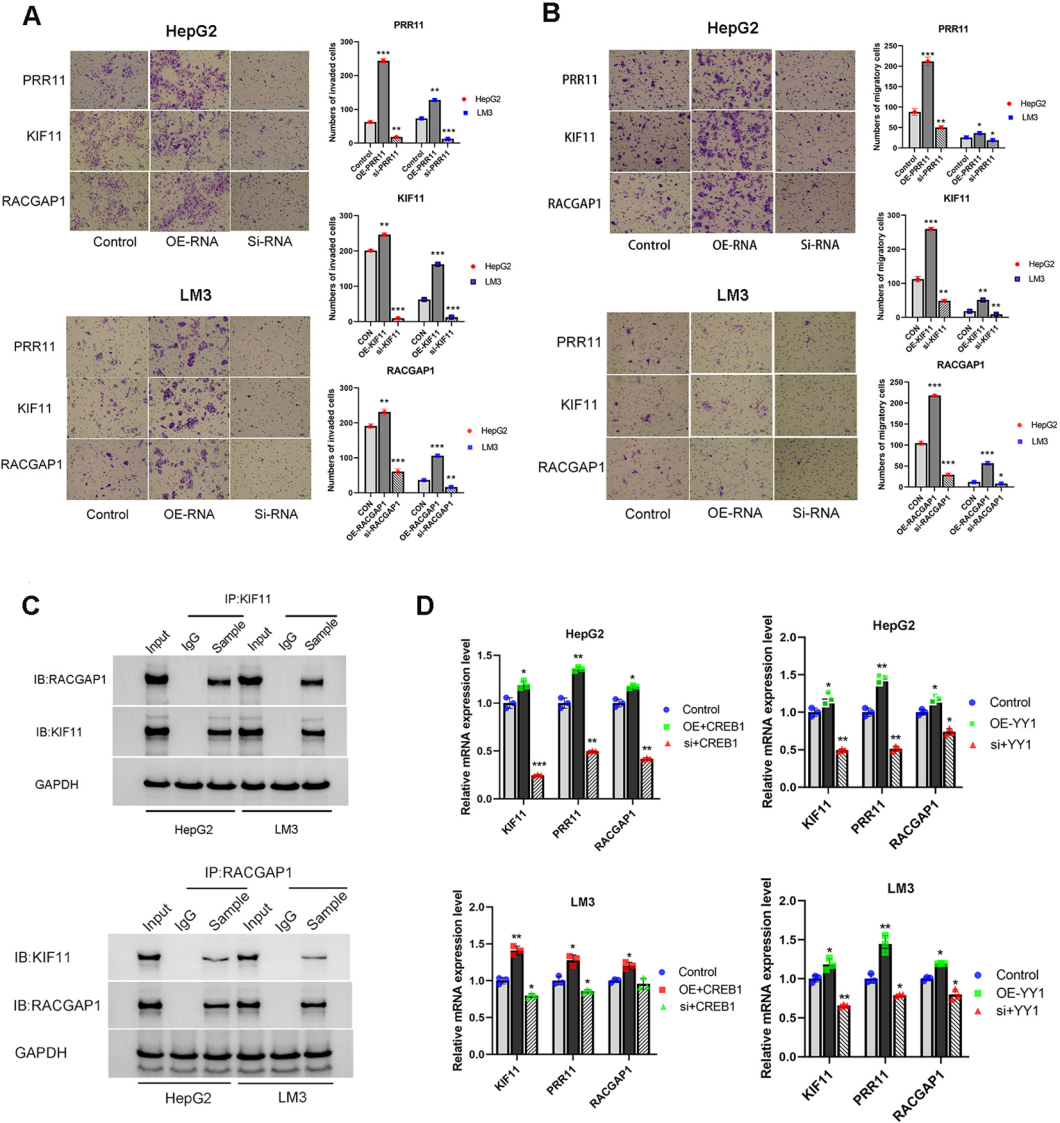
High expression of PRR11, KIF11, RACGAP1, YY1 and CREB1 promotes tumorigenic properties of HCC cells. **(A, B)** Migration and Matrigel invasion assays shows the effect of PRR11, KIF11, RACGAP1 expression levels on invasion and migration abilities of HepG2 and HCC-LM3 cells. Scale bar 50 µm. **(C)** The interaction between KIF11 and RACGAP1 was detected by immunoprecipitation in HepG2 and HCC-LM3 cells. **(D)** Effects of overexpression and silencing of YY1 and CREB1 on mRNA expression levels of PRR11, KIF11 and RACGAP1 in HepG2 and HCC-LM3 cells. *P<0.05, **P<0.01, ***P<0.001.

Transwell assays were performed to investigate the effects of PRR11, KIF11 and RACGAP1 on the invasion and migration of HCC cells. Our results revealed that overexpression of PRR11, KIF11 and RACGAP1 can enhance the invasion and migration of HepG2 and HCC-LM3 cells. Silencing PRR11, KIF11 and RACGAP1 attenuated the invasion and migration ability of HepG2 and HCC-LM3 cell (**Figures 13A, B**). These results confirmed the role of PRR11, KIF11 and RACGAP1 in promoting proliferation, invasion and migration of hepatocellular carcinoma.

CO-IP was used to confirm the protein association between KIF11 and RACGAP1 in HepG2 and HCC-LM3 cells (**Figure 13C**). Previous speculation was confirmed by the CO-IP results, which indicated that a close relationship exists between model factors. And this close relationship plays a synergistic role in promoting the development of PVTT.

Considering the low protein expression of SUZ12 in HCC tissues and HCC cell lines. Transcription factors YY1 and CREB1 were selected for subsequent experiments. After transient overexpression and silencing of YY1 and CREB1, the changes of model factors PRR11, KIF11 and RACGAP1 were detected. The results indicated that overexpression of YY1 and CREB1 could up-regulate the mRNA expression of PRR11, KIF11 and RACGAP1, while YY1 and CREB1 silencing could decrease the mRNA expression of PRR11, KIF11 and RACGAP1 (**Figure 13D**). This result validated our hypothesis that core factors share common transcription factors.

## Discussion

The present study entailed the systematic characterization of PVTT-associated biological characteristics in patients with HCC through the integration of scRNA-seq and bulk RNA-seq datasets. These approaches revealed that PVTT cells were characterized by stromal activation, tumor proliferation, and lower levels of immune cell infiltration, thus presenting with immune desert and immune rejection-related phenotypes.

Genes differentially expressed between PT and PVTT samples have previously been compared, revealing that the differences between these sample types are associated with cancer stem cells, extracellular stroma tissues, and immune cell disorders within the TME (40–42). Here, PVTT cells were found to exhibit the activation of processes such as the DNA repair, E2F target, mitotic spindle, G2M checkpoint, cell cycle, Hippo, Wnt, and TGF-β pathways. Core PVTT genes were closely associated with cells in the G2M phase of the cell cycle, and a positive correlation between the expression of these genes and RNAsi was observed. Together, these results suggest that PVTT cells present with active tumor proliferation closely associated with tumor stem cell function. There were also findings consistent with stromal activation including angiogenesis, EMT, and low levels of immunoinflammatory features including low inflammatory scores, low anti-inflammatory scores, and low antigen presentation scores in these PVTT samples. These results align well with past results. PT samples in cluster 1, in contrast, presented with an inflammatory pattern that was characterized by the activation of immune-inflammatory biological processes, including the complement, coagulation, and interferon-gamma pathways, together with higher ferroptosis, necroptosis, antigen presentation, and anti-inflammatory scores.

While PVTT primarily occurs in advanced HCC patients with clinical stage IIIB-IV disease, these analyses revealed that tumor cells with PVTT-like characteristics were present even in PT samples from individuals with early-stage HCC (clinical Stage I-II). This may account for the fact that some patients with HCC develop portal metastases even though they present with earlier clinical staging. PVTT-derived tumor cell developmental trajectories also exhibited some overlap with tumor cells with higher clinical staging, and there were significant differences from those of tumor cells with lower clinical staging, supporting the greater malignancy of PVTT tumor cells.

In past studies, PVTT patients have been separated into non-proliferative and proliferative types based on genomic profiling results (43), the mutational landscape, and key pathways associated with HCC development and progression. HBV-associated tumors tended to exhibit aggressive growth, more frequent vascular invasion, poor differentiation, and higher grades. Tumors of this type were characterized by greater chromosomal instability and enriched abnormal epigenetic signatures. These results align well with the present findings that core PVTT-associated genes were related to higher levels of CNV and somatic mutations, with high TMB and MSI scores being linked to the elevated expression of these core factors. This is particularly relevant as the analyzed PVTT samples were from Chinese patients, and most HCC patients in China are infected with HBV. Non-proliferative PVTT, in contrast, is less frequent in China and tends to be HCV- or alcohol-related, with lower AFP levels, better cell differentiation, and lower levels of aggression (44).

To determine how characteristic PVTT-related genes influence the biology of PVTT, 9 core genes were screened. These genes were found to include a transcription factor (MYBL2), cell cycle regulator (PRR11), cell division-related proteins (TUBA1B), kinases (PLK1, TRIP13), and RNA-binding proteins (MCM2, LMNB1, RACGAP1, KIF11). Through single-cell analyses, these genes were found to be expressed in eight cell types that included hepatocytes, fibroblasts, T cells, macrophages, and endothelial cells. These characteristic PVTT-related genes may influence the proliferation and invasivity of HCC cells, while also regulating these other cell types to control immunity and activate the tumor-associated stroma. Protein interactions for these core genes revealed that 8 of the genes exhibited interactive relationships, reflecting the closely associated roles that these core genes play in the promotion of HCC progression.

Patients were further clustered according to the expression patterns for 9 core genes, ultimately yielding three clustering patterns. In prognostic patterns, cluster B was associated with a pronounced survival advantage, whereas cluster C was linked to worse survival outcomes. GSVA and immune infiltration analyses revealed that these clusters were associated with distinct TME cell infiltration characteristics. Specifically, clusters A and B exhibited immune exclusion phenotypes with stromal activation and infiltration by innate immune cells, while cluster C exhibited an immune desert phenotype with characteristic immunosuppression. This aligns well with the results of prior studies in which immune characteristics were associated with patient prognosis (45, 46). This also suggests that expression differences for these core genes in PVTT patients play an important role in shaping TME heterogeneity at the individual level. While patients in cluster A exhibiting the immune rejection phenotype and survival disadvantages ultimately survived, most cluster B patients exhibiting pronounced survival advantages died. Additional analyses suggested that cluster A tumors may be more readily recognized by the immune system, activating more effective antitumor immunity, potentially accounting for the better prognostic outcomes for these patients.

In an effort to better account for the heterogeneous features of HCC patients, a scoring system was devised to assess the genetic characteristics of different patients with HCC. These analyses revealed that the cluster C pattern, which was characterized by immunosuppressive phenotypes, also presented with higher risk scores as compared to clusters A and B, which were instead characterized by immune exclusion phenotypes. These risk scores offered value as a tool to assess patient clinicopathological characteristics including tumor differentiation, clinical stage, histological subtype, molecular subtype, genetic variation, MSI status, and tumor mutation load. Strikingly, these risk scores were independent predictors of patient survival and could be used to predict adjuvant chemotherapy efficacy and patient responses to PD-1/CTLA4 immunotherapy. In summary, these risk scores can be utilized for the comprehensive evaluation of the risk of PVTT formation and associated changes in the infiltration of the TME in patients with HCC while providing further insight into tumor immunophenotyping that can more effectively guide clinical practice.

The results of MTT, colony formation assay, invasion and migration assay indicated that the model factors PRR11, KIF11 and RACGAP1 played an important role in promoting the progression of HCC. CO-IP assay was used to confirm the protein-protein interaction between KIF11 and RACGAP1. In addition, the common transcription factors YY1 and CREB1 predicted by bioinformatics methods were overexpressed and silencing, the mRNA expressions of model factors PRR11, KI and RACGAP1 were also observed to have corresponding changes. This result confirms the synergistic role of model candidate factors and transcription factors as a whole in promoting HCC progression.

## Conclusion

These analyses offer detailed insights into the biological features of PVTT in patients with HCC and the corresponding changes in the TME associated with this complication. Differences in key genes associated with PVTT were found to be closely associated with the heterogeneity and complexity of the TME in individual patients. Comprehensive analyses of patterns of gene expression such as this will provide detailed insight into cellular infiltration of the TME in patients with HCC, thereby guiding the design of more effective approaches to immunotherapy.

## Data Availability

The original contributions presented in the study are included in the article/**Supplementary Material**. Further inquiries can be directed to the corresponding authors.

## Supplementary material

Supplementary Figure 1
**(A)** Expression patterns for the top 30 marker genes in the three indicated cell clusters. **(B)** The relationship between the expression levels for 9 genes and the survival of HCC patients. **(C)** Survival risk plots for patients in the ICGC dataset. **(D)**The relationship between the levels of KIF11, RACGAP1, or RPR11 expression and the expression of other genes.

Supplementary Figure 2
**(A)** Protein interactions and weight relationships among 7 transcription factors. **(B)** Risk score distributions for different clinical subtypes of patients included in the ICGC cohort. **(C)** Responses to immunotherapy for patients with the indicated risk scores from the Melanoma Treatment study (GSE78220) and the Renal Cancer Treatment Study (GSE67501). **(D)** Bar graph of IHC scores of PRR11, KIF11, RACGAP1, YY1, CREB1, and SUZ12 in HCC tissues and adjacent para-tumor tissues.

Supplementary Figure 3 Electrophoretic gels and blots of Western blotting

Supplementary Figure 4 The original ethical review approval document
