# Supplementary figures and images for: Single-cell and bulk transcriptomic datasets enable the development of prognostic models based on dynamic changes in the tumor immune microenvironment in patients with hepatocellular carcinoma and portal vein tumor thrombus
Source: Front Immunol. 2024 Oct 28;15:1414121. doi: 10.3389/fimmu.2024.1414121 (PMC11550977; doi:10.3389/fimmu.2024.1414121)

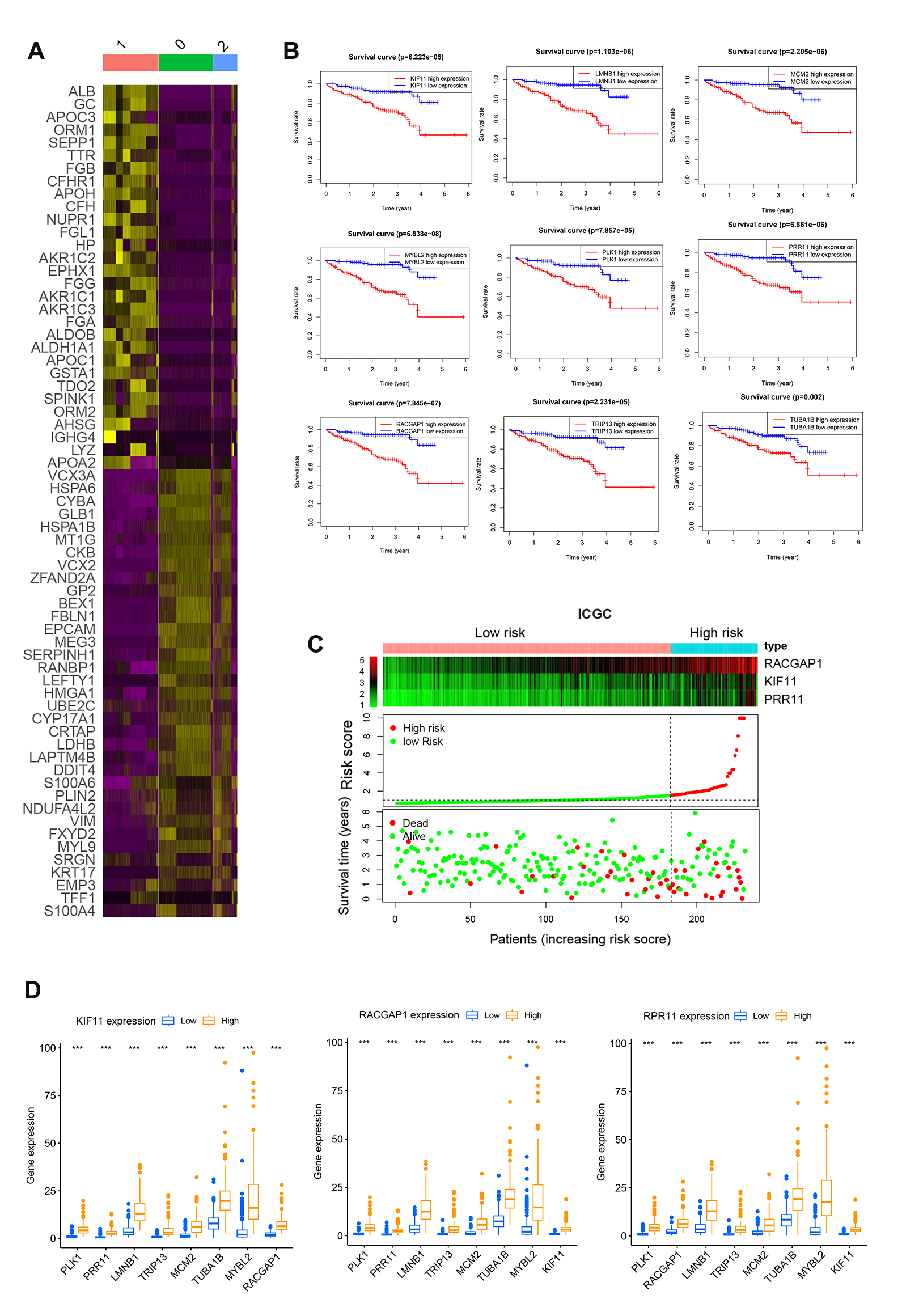

Supplement: Supplementary Figure 1 — (A) Expression patterns for the top 30 marker genes in the three indicated cell clusters. (B) The relationship between the expression levels for 9 genes and the survival of HCC patients. (C) Survival risk plots for patients in the ICGC dataset. (D)The relationship between the levels of KIF11, RACGAP1, or RPR11 expression and the expression of other genes. [file Image1.jpeg]

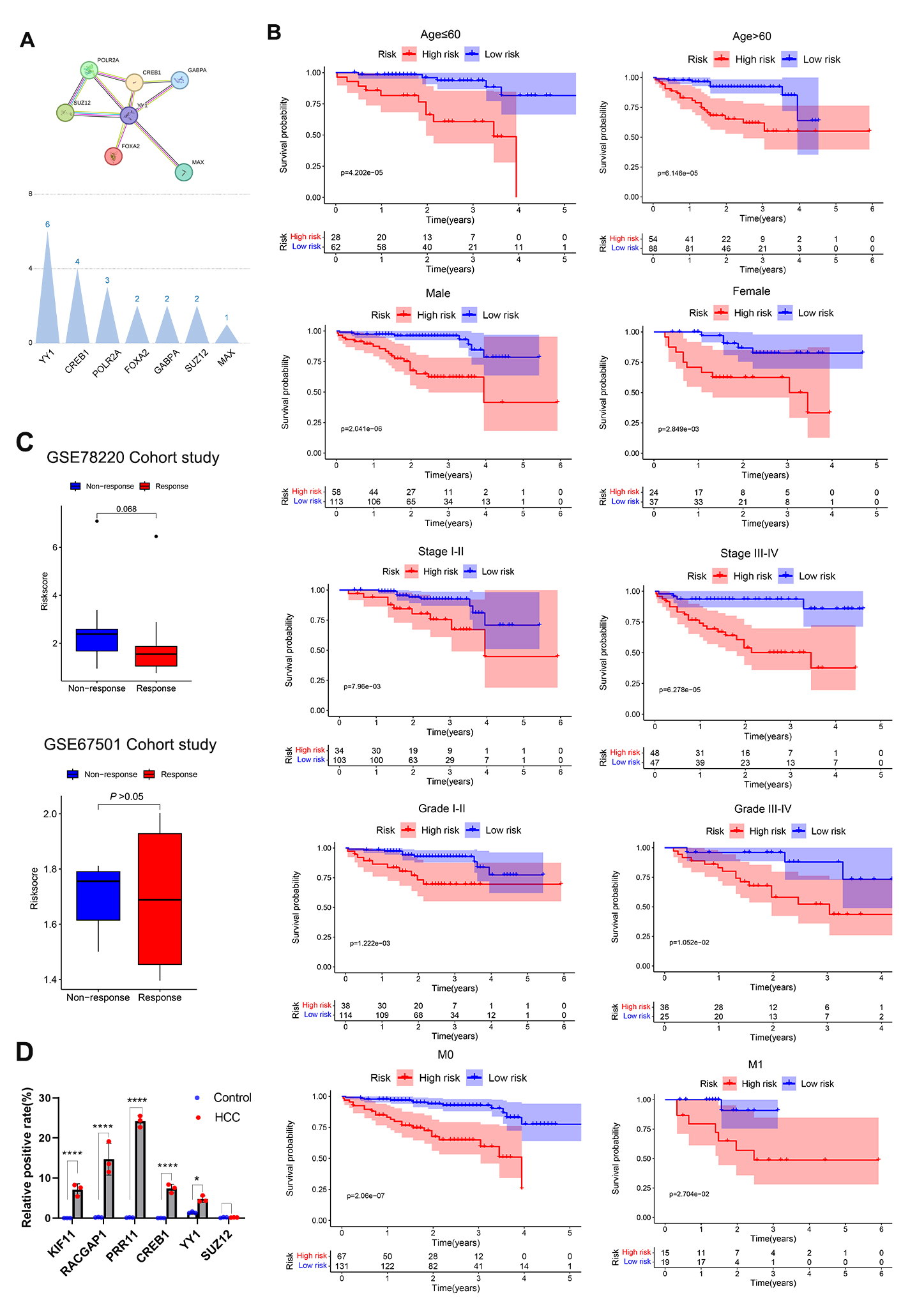

Supplement: Supplementary Figure 2 — (A) Protein interactions and weight relationships among 7 transcription factors. (B) Risk score distributions for different clinical subtypes of patients included in the ICGC cohort. (C) Responses to immunotherapy for patients with the indicated risk scores from the Melanoma Treatment study (GSE78220) and the Renal Cancer Treatment Study (GSE67501). (D) Bar graph of IHC scores of PRR11, KIF11, RACGAP1, YY1, CREB1, and SUZ12 in HCC tissues and adjacent para-tumor tissues. [file Image2.jpeg]
