# Supplementary material for: Single-cell and bulk transcriptomic datasets enable the development of prognostic models based on dynamic changes in the tumor immune microenvironment in patients with hepatocellular carcinoma and portal vein tumor thrombus
Source: Front Immunol. 2024 Oct 28;15:1414121. doi: 10.3389/fimmu.2024.1414121 (PMC11550977; doi:10.3389/fimmu.2024.1414121)

Supplementary Table 1. List of antibodies

Antigens	Species antibodies raised in	Dilution	Supplier
KIF11	Rabbit, polyclonal	1:1000(WB) 1:4000(IHC)	#ab254298, Abcam, Shanghai, China
RACGAP1	Rabbit, polyclonal	1:1000(WB) 1:200(IHC)	#13739-1-AP, Proteintech, Wuhan, China
CREB1	Rabbit, polyclonal	1:1000(WB) 1:100(IHC)	#bsm-34317R, Bioss, Beijing, China
PRR11	Rabbit, monoclonal	1:5000(WB) 1:200(IHC)	#PH16225S, Abmart , Shanghai, China
YY1	Rabbit, polyclonal	1:2000(WB) 1:200(IHC)	#bsm-52349R, Bioss, Beijing, China
SUZ12	Rabbit, polyclonal	1:1000(WB) 1:1000(IHC)	#EPR26230-82, Abcam, Shanghai, China
GAPDH	Rabbit, monoclonal	1:5000(WB)	#00143439, Proteintech, Wuhan, China

Supplementary Table 2 The primers for qRT-PCR

Target gene	Forward primer	Reverse primer
GAPDH	5'- CAGGAGGCATTGCTGATGAT -3'	5'- GAAGGCTGGGGCTCATTT -3'
KIF11	5'- GAAGACAATAAAGCAGACCC -3'	5'- TTTCCTCCAAAGCACAGA -3'
RACGAP1	5'- CTGAGGCTGACTGCGAAAA -3'	5'- TTGCTGCTGGATGGTTGG -3'
PRR11	5'- ATCTGCGGAAACTGCTTA -3'	5'- TTGGGCTTCTAGGGTGAG -3'
CREB1	5'- AGTGCCCAGCAACCAAGT -3'	5'- TTCCCTGTTCTTCATTAGACG -3'
YY1	5'- GGAATACCTGGCATTGACC -3'	5'- GCCGAGTTATCCCTGAACAT -3'
SUZ12	5'- AAAGAGCAACATGGGAGA -3'	5'- AAGAGGTTTGGCAATAGG -3'

Supplementary Table 3 si-RNA oligo for KIF11, RACGAP1, PRR11, CREB1, YY1 and SUZ12

Target gene	Sense	Antisense
KIF11-1	5'-UCAGAUUAUGCUCCAAGAUTT-3'	5'-AUCGAGGACCAUCAUCAUCTT-3'
KIF11-2	5'-UAGAAAGAGCCUUUGCCCATT-3'	5'-UGGGCAAAGGCUCUUUCUATT-3'

KIF11-3	5’-CAAGUACUUUGUAGCCAAATT-3’	5’-UUUGGCUACAAAGUACUUGTT-3’
RACGAP1-1	5’-CCUCUUGCCACUAGAAAUUTT-3’	5’-AAUUUCUAGUGGCAAGAGGTT-3’
RACGAP1-2	5’-CGUCUAGCCACUAGACAUUTT-3’	5’-AAUUUCUAGUCGCAUCAGCTT-3’
RACGAP1-3	5’-CUCUACGCCAUUAGAAUUATT-3’	5’-UUUAACUAGUCGCAUCGACTT-3’
PRR11-1	5’-GCCUGCAAACAUUAACCAUTT-3’	5’-AUGGUUAAUGUUUGCAGGCTT-3’
PRR11-2	5’-CUCUGCAAUCAUUAACGAUTT-3’	5’-UUGCUUAAUGAUUGCAGGGTT-3’
PRR11-3	5’-GCCUUCAAUCAUUAAACAATT-3’	5’-GUGGAUAAUGCUUGCAGGATT-3’
CREB1-1	5’-GCCUCUGGAGACGUACAAATT -3’	5’-UUUGUACGUCUCCAGAGGCTT-3’
CREB1-2	5’-GGACCUUUACUGCCACAAATT-3’	5’-UUUGUGGCAGUAAAGGUCCTT-3’
CREB1-3	5’-GCAGACAGUUCAAGUCCAUTT-3’	5’-AUGGACUUGAACUGUCUGCTT-3’
YY1-1	5’-GAAGAUGAUGCUCCAAGAATT -3’	5’-UUCUUGGAGCAUCAUCUUCTT-3’
YY1-2	5’-CCAAACAACUGGCAGAAUUTT-3’	5’-AAUUCUGCCAGUUGUUGCGTT-3’
YY1-3	5’-UCAGUCAACUAACCUGAAATT-3’	5’-UUUCAGGUUAGUUGACUGATT-3’
SUZ12-1	5’-CUCUGCCAUAGCAGAUUUATT-3’	5’-UAAAUCUGCUAUGGCAGAGTT-3’
SUZ12-2	5’-GCUGCCUCCAUUCGAAACATT-3’	5’-UGUUUCGAAUGGAGGCAGCTT-3’
SUZ12-3	5’-CAAGCCUGGUUCAGUUAAATT-3’	5’-UUUAACUGAACCAGGCUUGTT-3’

Supplementary Table 4 The primer of KIF11, RACGAP1, PRR11, CREB1, YY1 and SUZ12 for synthesizing cDNA for Plasmid construction.

**Target gene**	**primer**
KIF11-F	5’- GCTTGGTACCGAGCTCGGATCCGCCACCATGACCAAGGATTCTGGTGC CCAGTTCCACG-3’
KIF11-R	5’-TGCTGGATATCTGCAGAATTCTTAATCTGAGGCCGCTCTGTTGCACCGC GCAGGCCGAC-3’
CREB1-F	5’- GCTTGGTACCGAGCTCGGATCCGCCACCATGACCATGGAATCTGGAGC CGAGAACCAGC-3’
CREB1-R	5’-TGCTGGATATCTGCAGAATTCTTAATCTGATTTGTGGCAGTAAAGGTCC TTAAGTGCTT-3’
RACGAP1-F	5’- GCTTGGTACCGAGCTCGGATCCGCCACCATGGATACTATGATGCTGAAT GTGCGGAATC-3’
RACGAP1-R	5’-TGCTGGATATCTGCAGAATTCTCACTTGAGCATTGGAGAAGCAAAAAAG TTGCCTTGTC-3’
SUZ12-F	5’-GCTTGGTACCGAGCTCGGATCCGCCACCATGGCGCCTCAGAAGCACGGC GGTGGGGGAG-3’
SUZ12-R	5’-TGCTGGATATCTGCAGAATTCTCAGAGTTTTTGTTTTTTGCTCTGTTTTGA AACCCCTG-3’
YY1-F	5’-CTTGGTACCGAGCTCGGATCCGCCACCATGGCCTCGGGCGACACCCTCTA CATCGCCACGGACGG-3’
YY1-R	5’-TGCTGGATATCTGCAGAATTCTCACTGGTTGTTTTTGGCCTTAGCATGTGT

	TAAGATGTGAGATT-3’
PRR11-F	5’- GCTTGGTACCGAGCTCGGATCCGCCACCATGCCGATGGATTCTGCAGC CGAGAACCTGC-3’
PRR11-R	5’-TGCTGGATATCTGCAGAATTCTTAATCTGATTACTGGGAGTAATGGTGC TTAAGTCCTT-3’

Supplementary Table 5 Abbreviations

Full name	Abbreviation
Area under the curve	AUC
Copy number variation	CNV
Cytotoxic Lymphocyte Antigen	CTLA
Cancer Treatment Response Portal	CTRP
Differentially expressed genes	DEGs
Epithelial-mesenchymal transition	EMT
False discovery rate	FDR
Genomics of Drug Sensitivity in Cancer	GDSC
Genomics of Drug Sensitivity in Cancer	GDSC
Gene Expression Omnibus	GEO
Gene Ontology	GO
Gene set variation analysis	GSVA
Hepatic arterial infusion chemotherapy	HAIC
Hepatocellular carcinoma	HCC
International Cancer Genome Consortium	ICGC
Immuno-Oncology Biological Research	IOBR
Kyoto Encyclopedia of Genes and Genomes	KEGG
Liver Hepatocellular Carcinoma	LIHC
Overall survival	OS
Principal component analysis	PCA
Principal components	PCs
Primary tumor	PT
Portal vein tumor thrombosis	PVTT
Receiver Operating Characteristic Curve	ROS
Transcatheter arterial chemoembolization	TACE
The Cancer Genome Atlas	TCGA
The Cancer Imaging Archive	TCIA
Transforming growth factor-β	TGF-β
Tumor microenvironment	TME

Supplementary Figure 3

Fig.12B

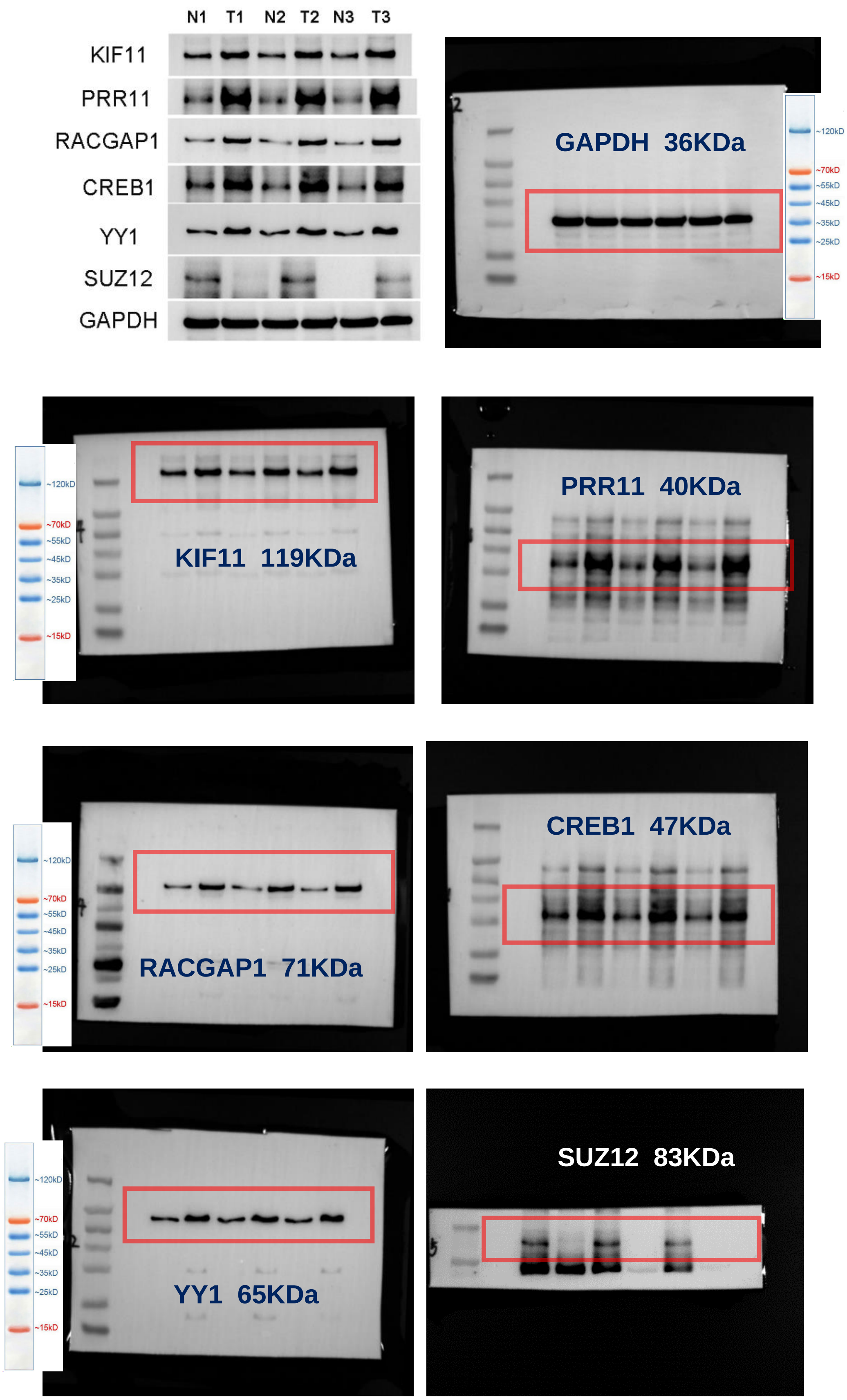

Fig.12D

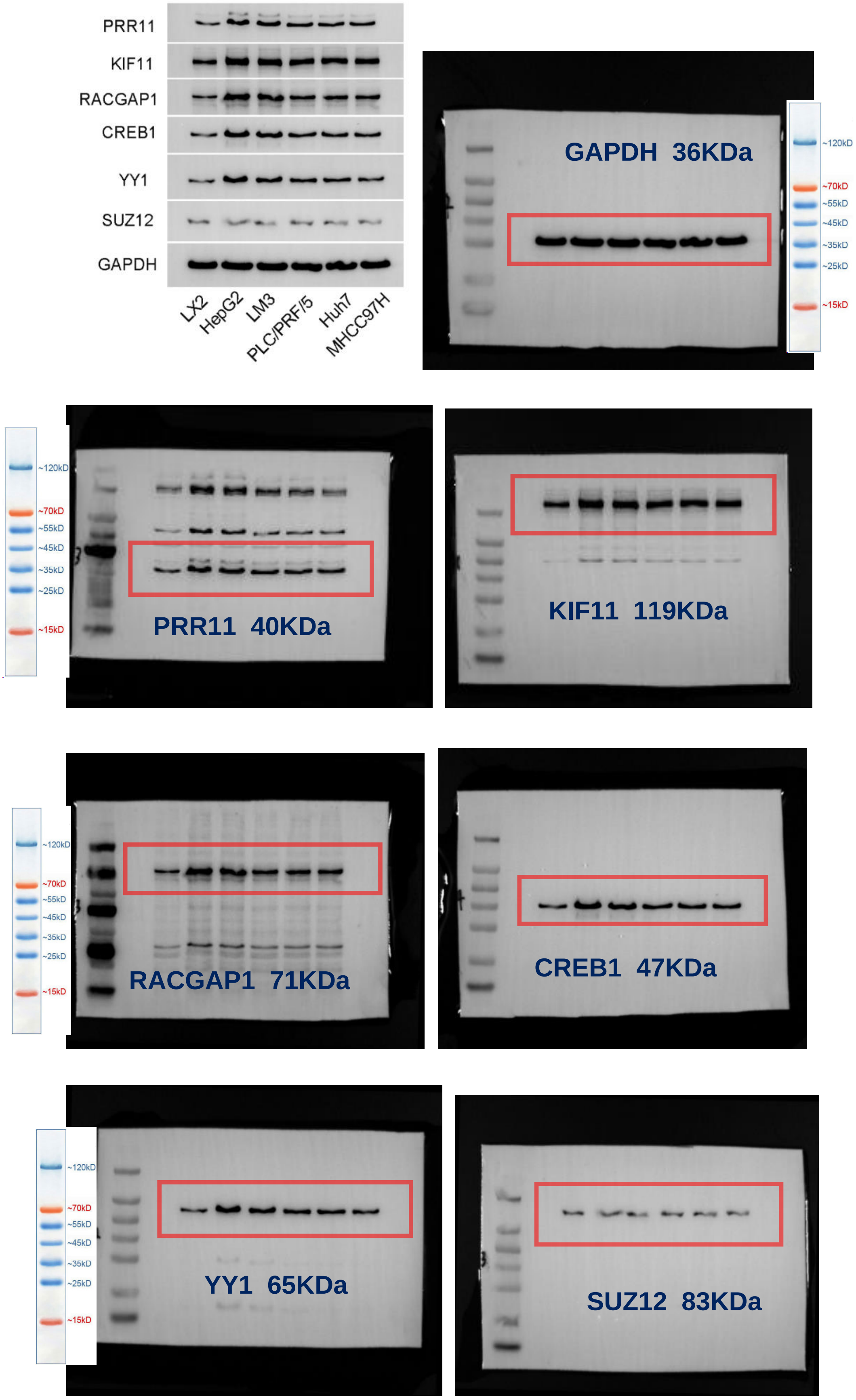
PRR11
KIF11
RACGAP1
CREB1
YY1
SUZ12
GAPDH
LX2
HepG2
LM3
PLC/PRF/5
Huh7
MHCC97H
GAPDH 36KDa
~120kD
~70kD
~55kD
~45kD
~35kD
~25kD
~15kD
PRR11 40KDa
KIF11 119KDa
RACGAP1 71KDa
CREB1 47KDa
YY1 65KDa
SUZ12 83KDa

Fig.13C

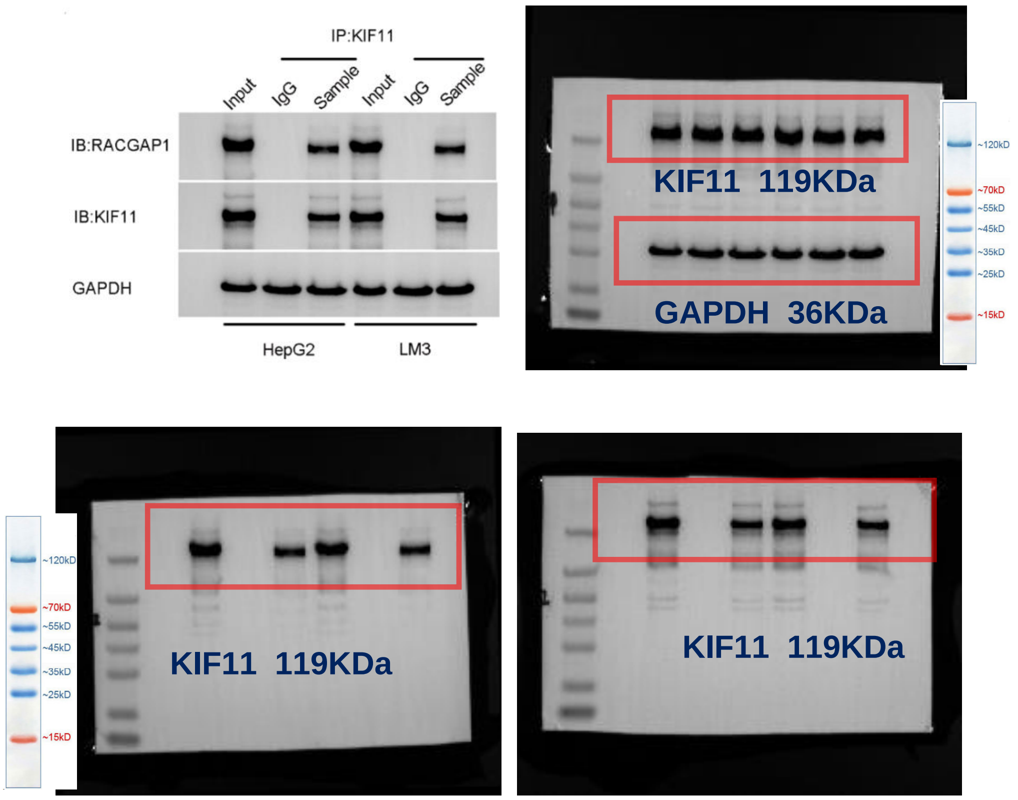

Fig.13C

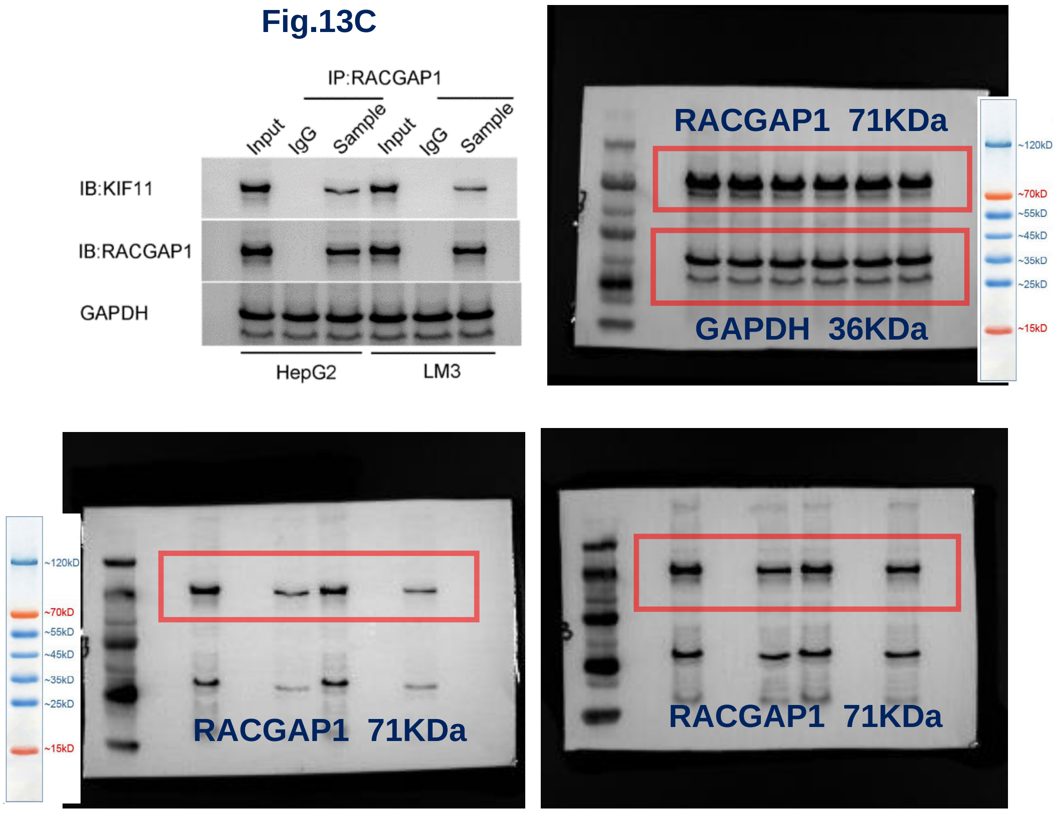

Supplement: Supplementary Figure 3 — Electrophoretic gels and blots of Western blotting [file DataSheet1.pdf]
